# Temporal Evolution of Bacterial Endophytes Associated to the Roots of *Phragmites australis* Exploited in Phytodepuration of Wastewater

**DOI:** 10.3389/fmicb.2020.01652

**Published:** 2020-07-17

**Authors:** Alberto Vassallo, Elisangela Miceli, Camilla Fagorzi, Lara Mitia Castronovo, Sara Del Duca, Sofia Chioccioli, Silvia Venditto, Ester Coppini, Donatella Fibbi, Renato Fani

**Affiliations:** ^1^Department of Biology, University of Florence, Sesto Fiorentino, Italy; ^2^G.I.D.A. SpA, Prato, Italy

**Keywords:** *Phragmites australis*, phytodepuration, wastewater, endophytes, antibiotic resistance, metal resistance

## Abstract

Improvement of industrial productions through more environment-friendly processes is a hot topic. In particular, land and marine environment pollution is a main concern, considering that recalcitrant compounds can be spread and persist for a long time. In this context, an efficient and cost-effective treatment of wastewater derived from industrial applications is crucial. Phytodepuration has been considered as a possible solution and it is based on the use of plants and their associated microorganisms to remove and/or transform pollutants. In this work we investigated the culturable microbiota of *Phragmites australis* roots, sampled from the constructed wetlands (CWs) pilot plant in the G.I.D.A. SpA wastewater treatment plant (WWTP) of Calice (Prato, Tuscany, Italy) before and after the CW activation in order to check how the influx of wastewater might affect the resident bacterial community. *P. australis* specimens were sampled and a panel of 294 culturable bacteria were isolated and characterized. This allowed to identify the dynamics of the microbiota composition triggered by the presence of wastewater. 27 out of 37 bacterial genera detected were exclusively associated to wastewater, and *Pseudomonas* was constantly the most represented genus. Moreover, isolates were assayed for their resistance against eight different antibiotics and synthetic wastewater (SWW). Data obtained revealed the presence of resistant phenotypes, including multi-drug resistant bacteria, and a general trend regarding the temporal evolution of resistance patterns: indeed, a direct correlation linking the appearance of antibiotic- and SWW-resistance with the time of exposure to wastewater was observed. In particular, nine isolates showed an interesting behavior since their growth was positively affected by the highest concentrations of SWW. Noteworthy, this study is among the few investigating the *P. australis* microbiota prior to the plant activation.

## Introduction

Plants and microorganisms have been living in association for a very long time. In fact, arbuscular mycorrhizal mutualism is believed to have had a key importance in the terrestrialization process and in the evolution and diversification of plant phototrophs (Selosse and Le Tacon, 1998; Heckman et al., 2001). Different microorganisms (bacteria and fungi) can establish (more or less) deep associations with plants; some of them exhibit an endophytic lifestyle, in that they colonize plant tissues internally, although a more specific definition of endophytes states that they are organisms which, at some moment of their life cycle, colonize the internal plant tissues without causing any type of harm to the host (Patriquin and Döbereiner, 1978). Potential endophytes often inhabit the surrounding soil, especially rhizosphere, from where they can enter plant tissues switching to an endophytic lifestyle. They may thus enter plant tissues through wounds, germinating radicles, emergence points of lateral roots or root elongation and differentiation zones (Reinhold-Hurek et al., 2006; Sturz et al., 2010). Once inside, bacteria adapt to different environmental conditions (e.g., pH, osmotic pressure, carbon source, and availability of oxygen) and overcome plant defense responses (Zeidler et al., 2004).

The plant host and the bacterial endophytes create a mutualistic interaction, with bacteria gaining nutrients and a niche to colonize (Sturz et al., 2010). Even though the exact role of endophytes within plant tissues has not been fully understood yet, it is well-established that in many cases endophytes are beneficial to plants (Schlaeppi and Bulgarelli, 2015; Wani et al., 2015). The most common functions observed for bacterial endophytes are (i) uptake of nutrients (e.g., N, P, S, Mg, Fe, and Ca; Duijff et al., 1999; Çakmakçi et al., 2006), (ii) biosynthesis of phytohormones promoting plant growth (Spaepen et al., 2007), (iii) 1-aminocyclopropane-1-carboxylate deaminase activity (ACC; Glick et al., 2007), (iv) nitrogen fixation (Doty et al., 2009), (v) prevention of pathogenic infections (Weller, 2007; Pérez-García et al., 2011), (vi) acceleration of seedling emergence (Hardoim et al., 2008), and (vii) tolerance to pollution and stresses (Ryan et al., 2008; Lugtenberg and Kamilova, 2014).

In the context of the present work, particularly important is the ability of plant-associated bacteria to increase tolerance to pollution and/or increase the ability of plants to detoxify polluted environments. Environmental pollution, especially water pollution, represents a concern of considerable prominence in the current society. In this regard, phytodepuration is the over-arching term for a group of technologies that utilizes plants and the associated rhizospheric microorganisms to remove and/or transform contaminants leached from soils/sediments and from used water streams (He et al., 2017; Saxena et al., 2020). It represents an environmental-friendly and a valuable solution for environmental cleanup, in particular for wastewater treatment, and it is popular because of its cost effectiveness, aesthetic advantages, and long-term applicability (Puvanakrishnan et al., 2019). In the present manuscript the term “phytodepuration” has been used to indicate specifically the remediation process regarding water and wastewater, rather than “phytoremediation,” which has a more general meaning, encompassing applications regarding, for example, soil remediation.

The constructed wetlands (CW) are engineered systems designed to mimic the self-purification processes of natural wetlands. For decades, CW have been successfully used for treating wastewater of different origins and have been identified as a sustainable wastewater management option worldwide (Wang et al., 2017), demonstrating their ability to eliminate diffuse pollutants from urban, rural, and industrial emissions. In literature, the effectiveness of the use of CW in the treatment of sewage containing heavy metals and high salinity is reported (Vymazal, 2011). This process is due to the interaction between plants, microorganisms, soil, and polluting substances (Zhou et al., 2013).

In CW, the rhizosphere is the mainly involved plant compartment, where multiple different physiochemical and biological processes occur (Stottmeister et al., 2003). The common reed *Phragmites australis* is one of the most employed plant species, because of its ability to flourish in marshy areas and swamps and the high detoxification and phytodepuration potential. Moreover, it is widely used to treat industrial wastewater containing heavy metals (Zhang et al., 2017). One peculiar characteristic of *P. australis* is that its internal environment is characterized by a relatively constant osmotic gradient determined by the downward transportation of Na^+^ from stems to roots (Vasquez et al., 2006). For this reason, *P. australis* is also well-adapted to salty ecosystems. In CW, vegetation is responsible for only a small amount of pollutant removal (0.02%; Zhang et al., 2017), while its main function is to provide additional oxygen and organic matter for microbial growth (Zhou et al., 2013). Indeed, microorganisms have been described as the main actors of pollutant removal in CW (Zhang et al., 2017). Phytodepuration has proved to effectively remove or neutralize hazardous environmental contaminants and it is predicted to have a growing application in the next years. However, this process presents some limitations, such as the toxic effects of pollutants on the growth and health of the plants (Glick, 2003). In fact, plant biomass is critical for phytodepuration (Germaine et al., 2010) and even hyperaccumulator plants, which can accumulate concentrations of toxic elements up to 100-fold higher than other plant species, usually exhibit a reduced growth. Also, phytodepuration may determine the accumulation of contaminants in plant tissues, which, in turn, is responsible for ecological and airborne exposure issues (Ho et al., 2012). In this scenario, rhizobacteria and endophytic bacteria can aid plants by supporting their growth (Tesar et al., 2002; Shaw and Burns, 2004; Chaudhry et al., 2005), reducing phytotoxicity effects, increasing pollutant uptake and removal (Glick and Stearns, 2011), reducing the release of toxic compounds into the atmosphere (Barac et al., 2004), removing contaminants and/or accumulating heavy metals (Germaine et al., 2010; Ho et al., 2012).

The experimental plant of Calice (Prato, Italy), managed by G.I.D.A. SpA, has therefore set itself as a goal to verify the action of this association in tertiary treatment of landfill leachate (LFL; Coppini et al., 2019).

The aim of this work was to characterize the cultivable bacterial communities associated to the roots of *P. australis* plants in Calice CW and to analyze their temporal dynamics before and after the activation of the plant for 22 months. This allowed the assessment of wastewater influx effect in shaping the composition of pre-existing bacterial communities. Moreover, bacteria isolated from roots were tested for their ability to grow in the presence of synthetic wastewater (SWW), along with their resistance against a panel of antibiotics commonly used to treat infections in humans. To the best of our knowledge, this work is among the few taking in consideration the bacterial composition of endophytes before the activation of CW, and likely the first regarding this issue in *P. australis*.

## Materials and Methods

### Site Description

*P. australis* plants were obtained from the CWs pilot plant managed by G.I.D.A. SpA and located at Calice Wastewater Treatment Plant (WWTP) in Prato, Italy. The CW of Calice was designed for the tertiary treatment of LFL. This CW is located downstream of a membrane bio-reactor (MBR) designed to pretreat a mixture of LFLs before their discharging in the main line of a full-scale WWTP, which treats both urban and industrial wastewater.

Constructed wetlands medium, used as substrate for the growth of *P. australis*, consists of four layers of gravel and sand; proceeding from the top to the bottom they are (thickness of layers and diameter range of particles are reported in brackets, respectively): gravel (20 cm; 5–10 mm) – sand (60 cm; 0.1–0.4 mm and 0.02–0.1 mm) – gravel (10 cm; 5–10 mm) – gravel (10 cm; 40–70 mm). CW implant was designed with two parallel lines, named “Line A” and “Line B,” respectively, with a total surface area of 1,620 m^2^. Each line is a two-stage subsurface flow system (SFS), consisting of a vertical system (SFS-v) followed by a horizontal one (SFS-h). The SFS-v of Line A is subdivided into four parallel separated tanks (SFS-v1, SFS-v2, SFS-v3 e SFS-v4), while the SFS-v of Line B is composed by two tanks (SFS-v5 e SFS-v6). Furthermore, both SFS-h lines are composed by three tanks, each one receiving the same hydraulic load. The maximum hydraulic load supplied to the entire system was 95 m^3^/day corresponding to a 1.9-day Hydraulic Retention Time for the horizontal stage (Coppini et al., 2019).

### Sampling and Isolation of Bacteria

Samples from the roots of *P. australis* were collected using sterile plastic bags and immediately transported to the laboratory for the subsequent processing. All procedures described hereinafter were carried out under sterile conditions to avoid external contaminations. Samples of three different specimens of *P. australis* growing in three different tanks were grouped and pooled before starting any procedure. Two pools were obtained from both SFS-v and SFS-h for each sampling campaign. 1 g of fresh tissue from each pool was surface-sterilized with 1% v/v HClO solution at room temperature to remove epiphytic bacteria and then washed three times with sterile water. Aliquots of 100 μL of water from the last wash were plated in triplicate as sterility controls. Subsequently, samples were homogenously pottered in a sterile mortar with the addition of 2 mL of 0.9% w/v NaCl sterile solution. Serial dilutions of tissue extracts were plated in triplicate on trypticase soy agar (TSA) medium (Biolife) and incubated at 30°C for 48 h. The total number of aerobic heterotrophic fast-growing bacteria of each sample was expressed as colony forming units per gram of roots (CFU/g), and it was determined as an average of three replicates. Isolated bacteria were name-coded according to the portion of the CW from whom they were isolated (V for the SFS-v and H for the SFS-h, respectively) and the pool of origin collected during the five samplings (1–2, 3–4, 5–6, 7–8, and 9–10 for the first, second, third, fourth, and fifth sampling, respectively).

### Amplification and Sequencing of 16S rRNA Gene

Polymerase chain reaction (PCR) were performed to amplify the 16S rRNA coding gene. 2 μL of colony thermal lysate were used as template for a PCR in 1X DreamTaq Buffer (Thermo Scientific) containing 200 μM of each dNTPs, 0.2 μM of primers P0 (5′-GAGAGTTTGATCCTGGCTCAG-3′) and P6 (5′-CTACGGCTACCTTGTTACGA-3′; Di Cello and Fani, 1996), 2 U of DreamTaq DNA Polymerase (Thermo Scientific) in a final volume of 25 μl. The PCR cycling for 16S rRNA gene amplification was 95°C for 3 min followed by 30 cycles of 95°C for 30 s, 55°C for 30 s, and 72°C for 1 min, then a final extension at 72°C for 10 min. A Bio-Rad T100 thermal cycler was used. Sequencing of 16S rRNA gene was performed by IGA Technology Services Srl (Udine, Italy).

### Taxonomic and Phylogenetic Analyses

Taxonomic affiliation of isolates was determined through the alignment of sequences to those of type strains downloaded from the ribosomal database project (RDP; Cole et al., 2014) using BioEdit (Hall, 1999). The obtained alignment was then used to build a phylogenetic tree through MEGA7 (Kumar et al., 2016), applying the Neighbor-Joining algorithm with a 1000-bootstrap resampling.

### Antibiotic Resistance Assay

Isolates were tested for their resistance against eight antibiotics (i.e., rifampicin, ampicillin, kanamycin, tetracycline, chloramphenicol, streptomycin, trimethoprim and ciprofloxacin) at six different concentrations (i.e., 1 – 10 – 25 – 50 – 100 – 150 μg/mL; Table 1). Bacteria were firstly grown overnight on TSA (Biolife) at 30°C, then a single colony was resuspended in 100 μL of 0.9 w/v NaCl sterile solution. The obtained suspensions were streaked on Mueller–Hinton II Agar (Biolife) plates supplemented with the tested antibiotics. Bacteria were also cultivated on the same medium in the absence of antibiotics, using these cultures as control to evaluate the presence of growth inhibition in presence of antibiotics. All plates were incubated at 30°C and growth performances were evaluated after 48 h. The minimal inhibitory concentration (MIC) value for each antibiotic was considered as the lowest concentration of the compound that inhibited visible growth of the tested isolate.

**TABLE 1:** Antibiotics used in this work.

Antibiotic	Class	Target
		
Ampicillin	Penicillins	Cell wall synthesis: inhibitor of D-Ala-D-Ala carboxypeptidase
Chloramphenicol	Phenicols	Ribosome: inhibitor of peptidyl transferase activity of 23S rRNA
Ciprofloxacin	Fluoroquinolones	Topoisomerases
Kanamycin	Aminoglycosides	Ribosome: inhibitor of 30S ribosomal subunit
Rifampicin	Ansamycins	DNA-dependent RNA polymerase
Streptomycin	Aminoglycosides	Ribosome: inhibitor of 30S ribosomal subunit
Tetracycline	Tetracyclines	Ribosome: it blocks the binding of aminoacyl-tRNAs
Trimethoprim	Diaminopyrimidines	DNA replication: inhibitor of dihydrofolate reductase

### Growth in Presence of Synthetic Wastewater

Growth of strains isolated from roots of *P. australis* in presence of SWW was assayed through the broth microdilution methods (Wiegand et al., 2008) using trypticase soy broth (TSB) medium (Biolife). The bacterial inoculum for the experiment was prepared by dissolving an isolated bacterial colony in 10 ml of TSB medium after 24 h-growth at 30°C on TSA. The inoculum was incubated overnight at 30°C under shaking. Upon incubation, absorbance at 600 nm was measured and adjusted to 0.1. The experiment was performed using 96-well plates. Each well contained 10 μL of bacterial inoculum, 80 μL of TSB medium and 10 μL of 10X, 20X, and 30X SWW, to reach the final concentration of 1X, 2X, and 3X, respectively. The composition of SWWs used for this assay is shown in Table 2. Growth performances in presence of SWW were evaluated calculating the ratio between the OD_600_ of cultures in presence of SWW (herein after indicated as OD_600SWW_) and OD_600_ of controls (i.e., bacteria grown in TSB lacking SWW). Bacterial isolates were considered sensitive to SWW when this parameter assumed values <0.7, while they were evaluated as resistant when it was >1.3.

**TABLE 2:** Composition of synthetic wastewaters (SWWs).

Compound	1X SWW	2X SWW	3X SWW
H_3_BO_3_	20	40	60
FeCl_2_ ⋅ 4H_2_O	15	30	45
Na_2_SeO_3_	0.03	0.06	0.09
NaCl	5,000	10,000	15,000

*Concentrations are expressed as mg/L.*

## Results

### Bacterial Counts

*P. australis* plants were sampled from the CW in Calice during a period of 22 months, spanning from March 2017 to December 2018; 5 samplings were conducted, with the first one (i.e., March 2017) performed before the activation of the CW (Table 3). This experimental strategy allowed us to compare the composition of the cultivable bacterial community associated to the *P. australis* roots before and after the beginning of wastewater influx. The titer of viable bacteria associated to roots was determined as described in section “Materials and Methods”. Data obtained revealed that there were no great differences between the CFU counts in SFS-v and SFS-h, exception for the third and fourth samplings in which CFU values were higher in SFS-h (Table 3).

**TABLE 3:** Bacterial counts in roots of *Phragmites australis* collected during the five samplings and meteorological conditions registered monthly.

	Samplings				
	
	1st (March 2017)	2nd (July 2017)	3rd (November 2017)	4th (June 2018)	5th (December 2018)
SFS-v (CFU/g)	4 × 10^6^	1 × 10^7^	1 × 10^6^	3 × 10^6^	6 × 10^5^
SFS-h (CFU/g)	5 × 10^6^	1 × 10^7^	5 × 10^6^	2 × 10^7^	1 × 10^6^
Average air temperature (°C)	13.8	26.4	10.6	23.5	7.4
Total precipitations (mm)	58.8	2.0	108.2	48.6	42.6

*Bacterial counts are expressed as colony forming units per gram (CFU/g) of roots, air temperature in Celsius degrees, total precipitations in millimeters.*

In general, bacterial load was quite constant during the experiment and fluctuations might be related to different factors, such as wastewater composition, frequency of raining, and/or seasonal variations. Indeed, it is likely that the weather exerted a main effect on bacterial growth since the highest bacterial loads were observed during summer (i.e., second and fourth samplings, respectively), when the higher temperatures probably facilitated bacterial growth and the poor precipitations probably caused a higher concentration of wastewater.

### Taxonomic Affiliation of Cultivable Bacteria

A total of 294 isolates (67, 49, 57, 68, and 53 from the first, second, third, fourth, and fifth sampling, respectively) were isolated from the *P. australis* roots. The attention was focused on bacteria isolated from this plant compartment because it has been reported that it is primarily involved in the depuration process (Riva et al., 2020). Each of the 294 isolates underwent a taxonomic characterization; to this purpose the amplification, sequencing, and analysis of 16S rRNA coding gene(s) were performed as described in section “Materials and Methods.”

**FIGURE 1:**
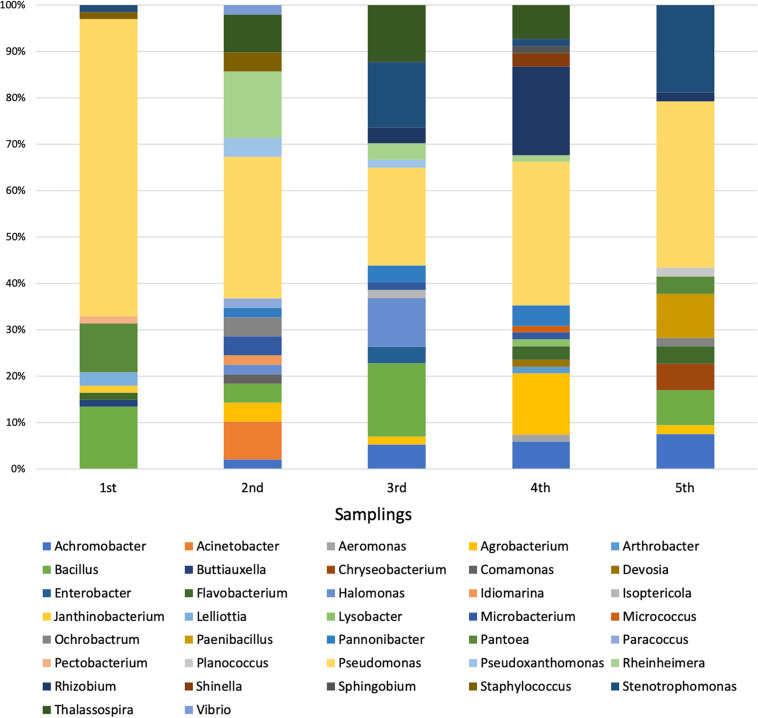
Distribution of bacterial genera during the five samplings.

Each sequence was submitted to Genbank and was assigned the accession number reported in Supplementary Table S1. The comparative analysis of each sequence with those available in databases allowed to split the 294 isolates into 37 different genera (Supplementary Figures S1–S30). The analysis revealed that 254 isolates were Gram-negative while 40 were Gram-positive bacteria. Moreover, a total of four different phyla were represented, with 246 belonging to *Proteobacteria* (59 *Alphaproteobacteria*, 14 *Betaproteobacteria*, and 173 *Gammaproteobacteria*), 8 to *Bacteroidetes* (all belonging to *Flavobacteriia* class), 33 to *Firmicutes* (all belonging to *Bacilli* class), and 7 to *Actinobacteria* (all belonging to *Actinobacteria* class). The most represented genus was *Pseudomonas*, whose members accounted for 37% of all isolates, as shown in Table 4. The abundance of *Pseudomonas* was not directly related to the activation of the CW, because it was the most represented genus even before the influx of wastewater (Figure 1).

**TABLE 4:** Number of bacterial isolates grouped for genus and sampling.

	Samplings						
	
	1st	2nd	3rd	4th	5th	Total	%
*Achromobacter*	–	1	3	4	4	12	4.08
*Acinetobacter*	–	4	–	–	–	4	1.36
*Aeromonas*	–	–	–	1	–	1	0.34
*Agrobacterium*	–	2	1	9	1	13	4.42
*Arthrobacter*	–	–	–	1	–	1	0.34
*Bacillus*	9	2	9	–	4	24	8.16
*Buttiauxella*	1	–	–	–	–	1	0.34
*Chryseobacterium*	–	–	–	–	3	3	1.02
*Comamonas*	–	1	–	–	–	1	0.34
*Devosia*	–	–	–	1	–	1	0.34
*Enterobacter*	–	–	2	–	–	2	0.68
*Flavobacterium*	1	–	–	2	2	5	1.70
*Halomonas*	–	1	6	–	–	7	2.38
*Idiomarina*	–	1	–	–	–	1	0.34
*Isoptericola*	–	–	1	–	–	1	0.34
*Janthinobacterium*	1	–	–	–	–	1	0.34
*Lelliottia*	2	–	–	–	–	2	0.68
*Lysobacter*	–	–	–	1	–	1	0.34
*Microbacterium*	–	2	1	1	–	4	1.36
*Micrococcus*	–	–	–	1	–	1	0.34
*Ochrobactrum*	–	2	–	–	1	3	1.02
*Paenibacillus*	–	–	–	–	5	5	1.70
*Pannonibacter*	–	1	2	3	–	6	2.04
*Pantoea*	7	–	–	–	2	9	3.06
*Paracoccus*	–	1	–	–	–	1	0.34
*Pectobacterium*	1	–	–	–	–	1	0.34
*Planococcus*	–	–	–	–	1	1	0.34
*Pseudomonas*	43	15	12	21	19	110	37.41
*Pseudoxanthomonas*	–	2	1	–	–	3	1.02
*Rheinheimera*	–	7	2	1	–	10	3.40
*Rhizobium*	–	–	2	13	1	16	5.44
*Shinella*	–	–	–	2	–	2	0.68
*Sphingobium*	–	–	–	1	–	1	0.34
*Staphylococcus*	1	2	–	–	–	3	1.02
*Stenotrophomonas*	1	–	8	1	10	20	6.80
*Thalassospira*	–	4	7	5	–	16	5.44
*Vibrio*	–	1	–	–	–	1	0.34
Total number of isolates	67	49	57	68	53	294	
Total number of genera	10	17	14	17	12	37	

*–: absence of isolates.*

Among the 37 genera, only 4 were exclusively present during the first sampling (i.e., *Buttiauxella*, *Janthinobacterium*, *Lelliottia*, and *Pectobacterium*), while 27 started being present from the second one on (i.e., *Achromobacter*, *Acinetobacter*, *Aeromonas*, *Agrobacterium*, *Arthrobacter*, *Chryseobacterium*, *Comamonas*, *Devosia*, *Enterobacter*, *Halomonas*, *Idiomarina*, *Isoptericola*, *Lysobacter*, *Microbacterium*, *Micrococcus*, *Ochrobactrum*, *Paenibacillus*, *Pannonibacter*, *Paracoccus*, *Planococcus*, *Pseudoxanthomonas*, *Rheinheimera*, *Rhizobium*, *Shinella*, *Sphingobium*, *Thalassospira*, and *Vibrio*). Hence, it is possible that the bacteria belonging to these 27 genera might derive from the wastewater, although it cannot be established whether they were present in wastewater with either urban or industrial origin. In addition to this, we cannot *a priori* exclude the possibility that they were already present in the pre-existing community (even though in low percentage) and that the presence of the wastewater might have exerted a selective pressure favoring their reproduction. In most cases, the phylogenetic trees showed a narrow taxonomic distribution of isolates, which clustered together in the same branch. For example, all *Acinetobacter* strains were phylogenetically close to *A. haemolyticus* (Supplementary Figure S1), all *Achromobacter* isolates formed a distinct cluster and were close to *A. spanius* (Supplementary Figure S5), all *Chryseobacterium* were related to *C. indoltheticum* (Supplementary Figure S7), all *Paenibacillus* belonged to the same cluster and were close to *P. tundrae* (Supplementary Figure S19), all *Pannonibacter* were affiliated to *P. phragmitetus* (Supplementary Figure S24), and all *Thalassospira* isolates formed a separate branch in the phylogenetic tree (Supplementary Figure S29). Moreover, *Rheinhemera* isolates distributed in close branches which included *R. aquimaris*, *R. pacifica*, and *R. nanhaiensis* (Supplementary Figure S22). On the contrary, a higher phylogenetic diversity was observed in the case of *Bacillus* (Supplementary Figure S4), *Enterobacteriales* (Supplementary Figure S10), *Pseudomonas* (Supplementary Figure S20), *Rhizobiales* (Supplementary Figure S23), and *Stenotrophomonas* (Supplementary Figure S28). However, with the exception of the bacteria belonging to the *Enterobacteriales* order, in the case of these genera the formation of distinct clusters was observed.

### Resistance Against Antibiotics

All 294 isolates were tested for their resistance against a panel of eight antibiotics used to treat human infections as described in section “Materials and Methods.” These compounds were chosen because they are representatives of diverse antibiotic classes and they are directed toward different cellular targets (Table 1). Data obtained are shown in Table 5 and Supplementary Table S2 and revealed that, overall, the most effective antibiotics were rifampicin, tetracycline and, above all, ciprofloxacin. On the contrary, the more tolerated antibiotic was ampicillin, especially in the case of *Pseudomonas* and *Stenotrophomonas* (Supplementary Table S2). Although resistant bacteria were isolated since the first sampling, a correlation between the time of exposure to the wastewater (i.e., earlier vs later samplings) and the increase of MIC values was observed for almost all antibiotics (Table 5 and Supplementary Table S2).

**TABLE 5:** Frequencies of minimal inhibitory concentration (MIC) values among bacterial isolates.

	MIC (μg/mL)	Samplings				
		
		1	2	3	4	5
Rifampicin	1	12	18	26	9	8
	10	26	28	9	21	20
	25	23	–	9	22	13
	50	–	–	11	1	5
	100	–	–	–	3	4
	150	–	–	–	–	2
	>150	–	–	–	–	–
Ampicillin	1	3	7	18	5	2
	10	1	8	1	1	3
	25	4	3	4	–	2
	50	2	7	6	5	5
	100	10	6	1	9	5
	150	1	4	2	3	5
	>150	39	8	23	33	30
Kanamycin	1	1	–	–	2	3
	10	50	25	31	16	27
	25	2	7	5	7	4
	50	3	3	2	14	1
	100	1	1	1	5	–
	150	1	2	1	–	–
	>150	1	6	15	12	17
Tetracycline	1	30	43	37	33	12
	10	28	4	8	19	31
	25	–	–	8	3	1
	50	–	–	1	–	–
	100	–	–	–	–	8
	150	–	–	–	–	–
	>150	–	–	–	1	–
Chloramphenicol	1	20	14	9	15	4
	10	8	10	17	5	9
	25	4	7	14	13	14
	50	4	7	8	6	14
	100	16	6	–	–	6
	150	2	2	2	–	1
	>150	7	–	4	17	4
Streptomycin	1	3	12	2	–	2
	10	32	13	24	16	10
	25	10	9	9	4	12
	50	10	3	1	5	–
	100	4	–	4	2	8
	150	1	–	–	9	–
	>150	3	7	14	20	20
Trimethoprim	1	12	15	8	–	5
	10	–	5	9	7	7
	25	1	3	2	2	7
	50	1	6	3	5	2
	100	2	7	15	4	5
	150	7	5	5	3	6
	>150	42	5	12	35	20
Ciprofloxacin	1	65	43	40	48	39
	10	–	3	14	8	5
	25	–	–	–	–	8
	50	–	–	–	–	–
	100	–	–	–	–	–
	150	–	–	–	–	–
	>150	–	–	–	–	–

*–: absence of isolates showing a specific MIC value.*

On one hand, tests with rifampicin, ciprofloxacin and tetracycline showed a progressive increase in the number of isolates having the highest MIC values going from the first to the fifth sampling. On the other one, although MIC values were not determined in the assayed conditions because isolates were able to grow even in presence of the highest concentration of antibiotic, in the case of kanamycin and streptomycin the number of isolates with MIC > 150 μg/mL increased during time. Finally, clear trends were not detected using ampicillin, chloramphenicol and trimethoprim: indeed, there were bacteria able to survive in the presence of the highest concentration since the first sampling and, also, the frequency of resistant isolates was not subjected to temporal variations.

According to the MIC breakpoints provided by the European Committee on Antimicrobial Susceptibility Testing (EUCAST – Breakpoint tables for interpretation of MICs and zone diameters; Version 10.0, 2020^1^), relatively to the antibiotics assayed in this work and limiting to the species reported by EUCAST, six isolates could be defined as multi-drug resistant strains, since they were resistant against at least three different antibiotics (Supplementary Table S2). In detail, two *Lelliottia* (V2R14 and H1R21) and two *Enterobacter* (H5R6 and H5R7) isolates were resistant to ampicillin, chloramphenicol, and ciprofloxacin; lastly, the two *Pantoea* isolates H9R2 and H9R15 that were resistant to ampicillin, chloramphenicol, trimethoprim, and ciprofloxacin.

### Growth in the Presence of SWW

The 294 isolated *P. australis* root-associated endophytes were also tested for their ability to grow in the presence of SWW containing B, Fe, and Se since these elements are critical for the WWTP studied in this work. Selection of bacteria able to grow efficiently in the presence of these compounds is of relevant interest because CW might be enriched with these more tolerant microorganisms, which, in turn, might increase the pollutant removal efficiency in wastewater. All bacterial isolates were assayed for their growth in TSB medium supplemented with three different concentration of SWW: 1X (i.e., a mix of H_3_BO_3,_ FeCl_2_ ⋅ 4H_2_O, Na_2_SeO_3_, and NaCl at the maximum concentrations allowed by law for sewer emission), and 2X and 3X in which TSB medium was supplemented with two- and threefold higher concentrations of 1X SWW, respectively.

**FIGURE 2:**
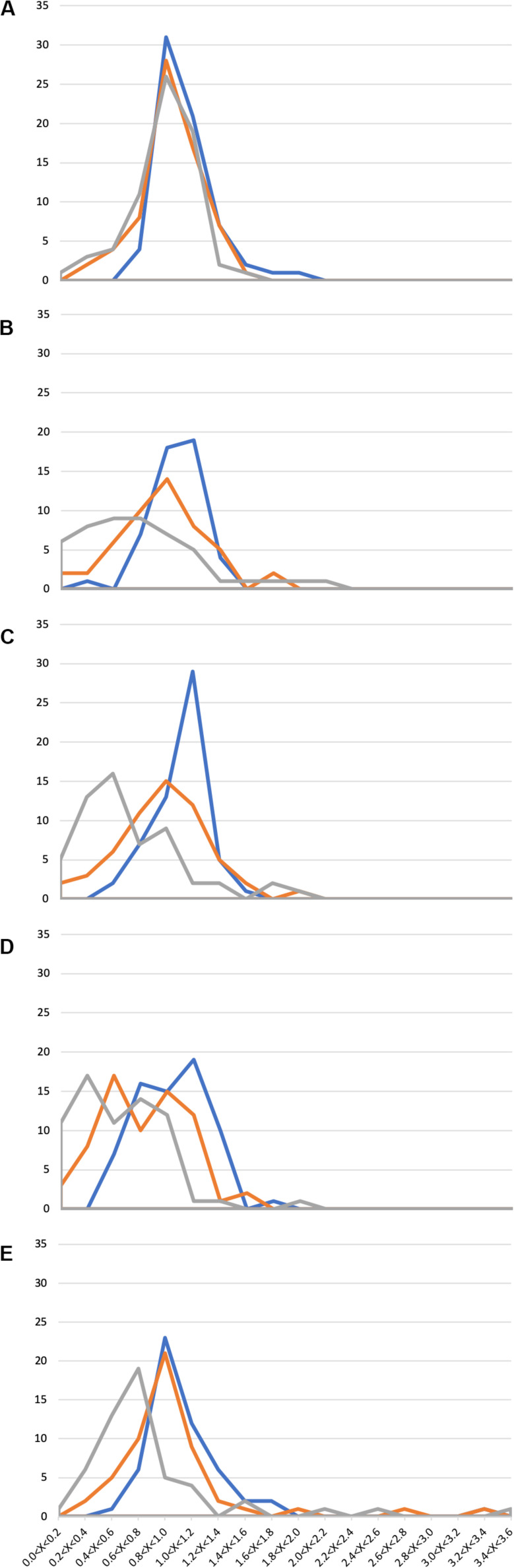
Growth of 294 *Phragmites australis* root-associated bacteria in the presence of synthetic wastewater (SWW). Ranges of OD_600SWW_/OD_600_ values are reported in the *x* axis, while the number of isolates is reported in the *y* axis. **(A)** 1st sampling; **(B)** 2nd sampling; **(C)** 3rd sampling; **(D)** 4th sampling; and **(E)** 5th sampling. Blue: 1X SWW; Orange: 2X SWW; Gray: 3X SWW.

In general, the presence of 1X SWW did not alter the growth of isolates, indicating that these endophytes can tolerate the presence of the tested compounds (Figure 2 and Supplementary Table S3). The analysis of data shown in Supplementary Table S3 and Figure 2 also revealed that 211 bacterial isolates were able to grow efficiently also in the presence of either 2X SWW or 3X SWW. Interestingly, eight isolates (i.e., V2R8, H3R17, H4R18, V6R1, V5R1, V8R24, H9R1, and H10R8), belonging to the genera *Bacillus*, *Planococcus*, *Pseudomonas*, and *Rheinheimera*, showed a positive correlation between growth and concentration of SWW: indeed, the higher the SWW concentration, the higher the growth of these bacteria. This finding suggests that these bacteria could represent good candidates for future applications and for improvements of phytodepuration efficiency and pollutant removal. Moreover, the isolate H9R16 deserves further investigations, since it showed the highest growth increase (about 350%) in presence of SWW. The analysis of the 16S rDNA phylogenetic tree revealed that it joined bacteria belonging to the *Bacillus gibsonii* species (Supplementary Figure S4), alkaliphilic bacteria exploited for production of alkaline proteases (Martinez et al., 2013; Deng et al., 2014).

Similarly to the case of antibiotic resistance, also in this assay a correlation between the time of exposure to wastewater in the CW and the appearance of more resistant isolates was highlighted. Indeed, a progressive increase of the number of isolates showing an augmented growth (measured as OD_600SWW_/OD_600_ ratio as described in section “Materials and Methods”) was observed from the first to the last sampling. For instance, several isolates with an OD_600SWW_/OD_600_ > 2.4 (Figure 2E) were detected only during the last sampling.

**FIGURE 3:**
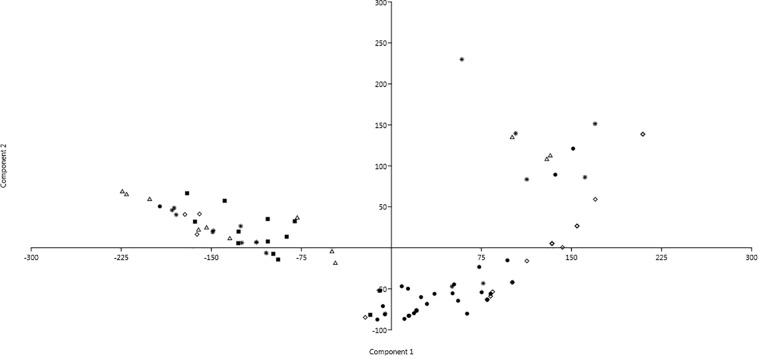
Principal component analysis (PCA) showing profiles of resistance against antibiotics and SWW of isolates belonging to the genus *Pseudomonas*. Dot: 1st sampling; Filled square: 2nd sampling; Triangle: 3rd sampling; Diamond: 4th sampling; and Star: 5th sampling.

Changings of resistance profiles along time, considering those against either antibiotics or SWW, were particularly clear in the case of isolates belonging to the genus *Pseudomonas*. As shown in Figure 3 by the mean of principal component analysis (PCA) performed with the PAST4 software (Hammer et al., 2001), the formation of two different main clusters was observed with the main part of isolates from the first sampling clustering independently from those isolated from all the other four samplings.

## Discussion

The aim of this work was the analysis of the composition, the phenotypic characterization, and the temporal dynamics of the cultivable microbiota associated to the roots of *P. australis* grown in the CW of Calice (Prato, Italy) before and after the activation of the CW.

The composition of root cultivable microbiota was determined through five samplings spanning from March 2017 to December 2018. We focused on cultivable bacterial communities because their isolation and characterization might permit the identification of strains particularly resistant to the antibiotics and/or to the compounds present in the wastewater. Hence, these strains could be used to construct a “synthetic” consortium that, in turn, might be exploited in pilot-experiments with the goal of increasing the phytodepuration efficiency of the plant. The taxonomic analysis was performed on 294 cultivable bacteria through the analysis of the 16S rRNA genes. Even though we are completely aware that the number of isolates could not be representative of the entire community, the analysis performed gave useful hints on the effect of the activation of CW on bacterial community composition. Data obtained revealed that the wastewater income exerted a shaping effect on the bacterial composition. Overall, 37 bacterial genera were disclosed and six of them were detected both before and after the activation of CW. Moreover, bacteria belonging to 27 different genera were detected only after the activation of the CW, while, on the contrary, 4 genera that were present at the beginning were not found in the following samplings. The most represented genus in all five samplings was *Pseudomonas*, which accounted for the 37% of all isolates.

As it might be expected, the effect of the wastewater income was exerted not only at the taxonomic level, but also at the phenotypic one. Each of the 294 bacterial isolates was assayed for its resistance against a panel of eight antibiotics belonging to different chemical classes and acting toward different cellular targets. This analysis was performed since it is known that WWTP are reservoirs of antibiotic resistance genes and/or resistant bacteria. Data obtained revealed the presence of several resistant (or multi-resistant) strains and, mostly important, that the number of antibiotic resistant isolates and the degree of antibiotic resistance increased over time, from the first to the last sampling. This strongly suggests that the wastewater income might generate a selective pressure favoring the growth of those isolates intrinsically resistant to antibiotics, even though it cannot be *a priori* excluded the possibility of horizontal gene transfer (HGT) events and/or the acquisition of resistance through mutations in chromosomal genes.

All the 294 isolates were assayed also for their ability to grow in SWW containing three different concentrations of B, Se, Fe, and NaCl. Analogously to the antibiotic resistance pattern, we detected a similar correlation between SWW exposure and the ability to grow in its presence. Interestingly, among isolates able to grow in the presence of these compounds, nine of them showed an increasing growth at the highest concentrations of SWW: these isolates will deserve a specific focus to identify the molecular mechanisms behind this intriguing behavior. Moreover, a PCA carried out on *Pseudomonas* strains, isolated from all the five samplings, furtherly suggested that the main event changing the resistance patterns against antibiotics and SWW was the activation of the plant (i.e., when conveyance of the permeate into the tanks occurred). The characterization of heavy metal resistant strains may be crucial to better understand the diffusion of antibiotic resistance genes in the environment. As a matter of fact, it has been reported that the occurrence of multiple heavy metal resistance markers is associated with the onset of antibiotic resistance (Wales and Davies, 2015; Wu et al., 2018; Zhu et al., 2019). This might be due to the co-localization of resistance genes against antibiotics and heavy metals in the same mobile genetic element(s) and, as a consequence, the accumulation of heavy metals in the environment can cause the selection of antibiotic resistant species. So, the dissemination of these heavy metal resistance genes represents an issue that should not be underestimated. Moreover, monitoring the presence of bacteria resistant to antibiotics and/or heavy metals specifically in WWTPs should be considered as a priority to contrast the spreading of multi-drug resistant (MDR) pathogens. Indeed, WWTPs represent hotspots for HGT events, because of the mixing of bacteria from diverse sources (e.g., households, hospitals, industries, etc.), the high bacterial densities, stressful conditions triggering SOS responses and presence of antibiotics at sublethal concentrations (Karkman et al., 2018). It must be also considered that although HGT occurring in WWTPs might not directly regard human pathogens, these could acquire resistance markers from harmless bacteria which act as vectors as soon as effluent is released in the environment (Manaia, 2017).

To deeply characterize this phenomenon, future work could take advantage of emulsion, paired isolation and concatenation PCR (epicPCR) as previously reported (Spencer et al., 2016; Hultman et al., 2018). Indeed, this kind of analysis could allow the “tagging” of resistance genes with phylogenetic markers, such as 16S rRNA gene, helping to compare these pairs in wastewater entering the WWTP and in effluents. However, this would be limited to target resistance markers with known sequence and for whom it is thereof possible to design specific primers.

## Conclusion

The experimental approach used in this work revealed that the cultivable bacterial community existing prior to the plant activation underwent fluctuations in terms of both taxonomy and resistance to antibiotics and SWW compounds. As it might be expected, the influx of wastewater exerted a selective pressure on the resident bacterial community, selecting and/or bringing bacterial strains progressively more resistant to SWW and/or antibiotics. We are completely aware that the analysis of the entire community (both cultivable and uncultivable) might give more detailed insights into the composition of the total community. In spite of this, only the selection of particular cultivable strains, i.e., more resistant to SWW and antibiotics, can permit the formulation of a synthetic bacterial community to improve the phytodepuration properties of *P. australis*.

## Data Availability Statement

The datasets generated in this study can be found in online repositories. The names of the repository/repositories and accession number(s) can be found at: https://www.ncbi.nlm.nih.gov/genbank/, MK110895, MK110946, MK110896, MK110920, MK110921, MK110947, MK110948, MK110922, MK110897, MK110949, MK110898, MK110950, MK110899, MK110959, MK110923, MK110925, MK110924, MK110960, MK110926, MK110945, MK110957, MK110927, MK110928, MK110929, MK110930, MK110931, MK110932, MK110900, MK110901, MK110902, MK110933, MK110934, MK110935, MK110958, MK110936, MK110937, MK110938, MK110939, MK110940, MK110941, MK110942, MK110943, MK110903, MK110904, MK110905, MK110906, MK110907, MK110908, MK110909, MK110910, MK110911, MK110912, MK110951, MK110913, MK110914, MK110915, MK110952, MK110916, MK110953, MK110917, MK110954, MK110918, MK110944, MK110961, MK110919, MK110955, MK110956, MK134509, MK134489, MK134488, MK134487, MK134486, MK134554, MK134508, MK134547, MK134496, MK138850, MK134502, MK134511, MK134551, MK134555, MK134559, MK134558, MK134549, MK138851, MK134553, MK134542, MK134557, MK134546, MK134544, MK134543, MK134541, MK134540, MK134510, MK134497, MK134490, MK134495, MK134494, MK134493, MK134499, MK134505, MK134552, MK134556, MK134545, MK134548, MK134500, MT165525, MK134507, MK134504, MK134503, MK134550, MK134501, MK134498, MK134506, MK134492, MK134491, MK134518, MK130934, MK130935, MK134524, MK134539, MK134538, MK134515, MK138852, MK134526, MK130907, MK130906, MK130937, MK134534, MK134533, MK138853, MK130915, MK130913, MK130910, MK130921, MK130917, MK134514, MK130914, MK134516, MK130911, MK134532, MK134531, MK134530, MK134528, MK134485, MK138854, MK130912, MK130908, MK130920, MK130932, MK130933, MK130936, MK134513, MK130922, MK130919, MK134521, MK134512, MK134537, MK134536, MK134535, MK134522, MK134519, MK134517, MK134529, MK134525, MK134523, MK134520, MK134527, MK130916, MK130931, MK130909, MK130923, MK130918, MK130945, MK130957, MK130901, MK130905, MK133358, MK138868, MK138869, MK138870, MK138872, MK138874, MK138875, MK130924, MK130928, MK138881, MK138862, MK130903, MK138867, MK138878, MK138861, MK130939, MK138863, MK130949, MK130953, MK130904, MK138876, MK138879, MK130926, MK130927, MK130929, MK138880, MK130930, MK138882, MK138883, MK138884, MK138885, MK138886, MK138887, MK130940, MK130941, MK130943, MK138889, MK130944, MK130902, MK130900, MK130946, MK138877, MK138855, MK138856, MK138857, MK138858, MK138859, MK138860, MK138864, MK138865, MK138866, MK138871, MK138873, MK130925, MK130942, MK130948, MK130950, MK130955, MK138888, MK130951, MK130952, MK130954, MK130956, MK130947, MT165526, MT165527, MT165547, MT165551, MT165553, MT165552, MT165528, MT165529, MT165530, MT165531, MT165532, MT165533, MT165534, MT165557, MT165554, MT165535, MT165536, MT165555, MT165558, MT165559, MT165561, MT165548, MT165562, MT165560, MT165549, MT165563, MT165564, MT165537, MT165538, MT165565, MT165539, MT165540, MT165566, MT165570, MT165569, MT165567, MT165571, MT165568, MT165578, MT165550, MT165541, MT165542, MT165543, MT165572, MT165544, MT165545, MT165573, MT165546, MT165574, MT165556, MT165575, MT165576, and MT165577.

## Author Contributions

RF, EC, and DF conceived the project. AV, EM, CF, and RF designed the experiments. AV, EM, CF, SV, SD, LC, and SC performed the experiments. RF supervised the experiments. AV, EM, and RF analyzed the results. AV wrote the original draft of the manuscript. AV, SD, LC, SC, CF, EM, EC, and RF reviewed and edited the manuscript. All authors read and approved the manuscript.

## Conflict of Interest

EC and DF were employed by G.I.D.A. SpA. The remaining authors declare that the research was conducted in the absence of any commercial or financial relationships that could be construed as a potential conflict of interest.

## Footnotes

1 http://www.eucast.org

## Supplementary Material

FIGURE S1–S30 Phylogenetic trees.

TABLE S1 List of bacteria isolated from roots of *Phragmites australis* and used in this work. Accession numbers of 16S rRNA gene partial sequences are reported.

TABLE S2 MIC (μg/ml) values of bacterial isolates described in this work.

TABLE S3 Growth of 294 bacterial isolates associated to *Phragmites australis* roots in TSB in presence of three different concentrations of synthetic wastewater (SWW).
