# Supplementary material for: Temporal Evolution of Bacterial Endophytes Associated to the Roots of Phragmites australis Exploited in Phytodepuration of Wastewater
Source: Front Microbiol. 2020 Jul 17;11:1652. doi: 10.3389/fmicb.2020.01652 (PMC7380131; doi:10.3389/fmicb.2020.01652)
Supplement: FIGURE S1–S30 — Phylogenetic trees. [file Table_4.DOCX]

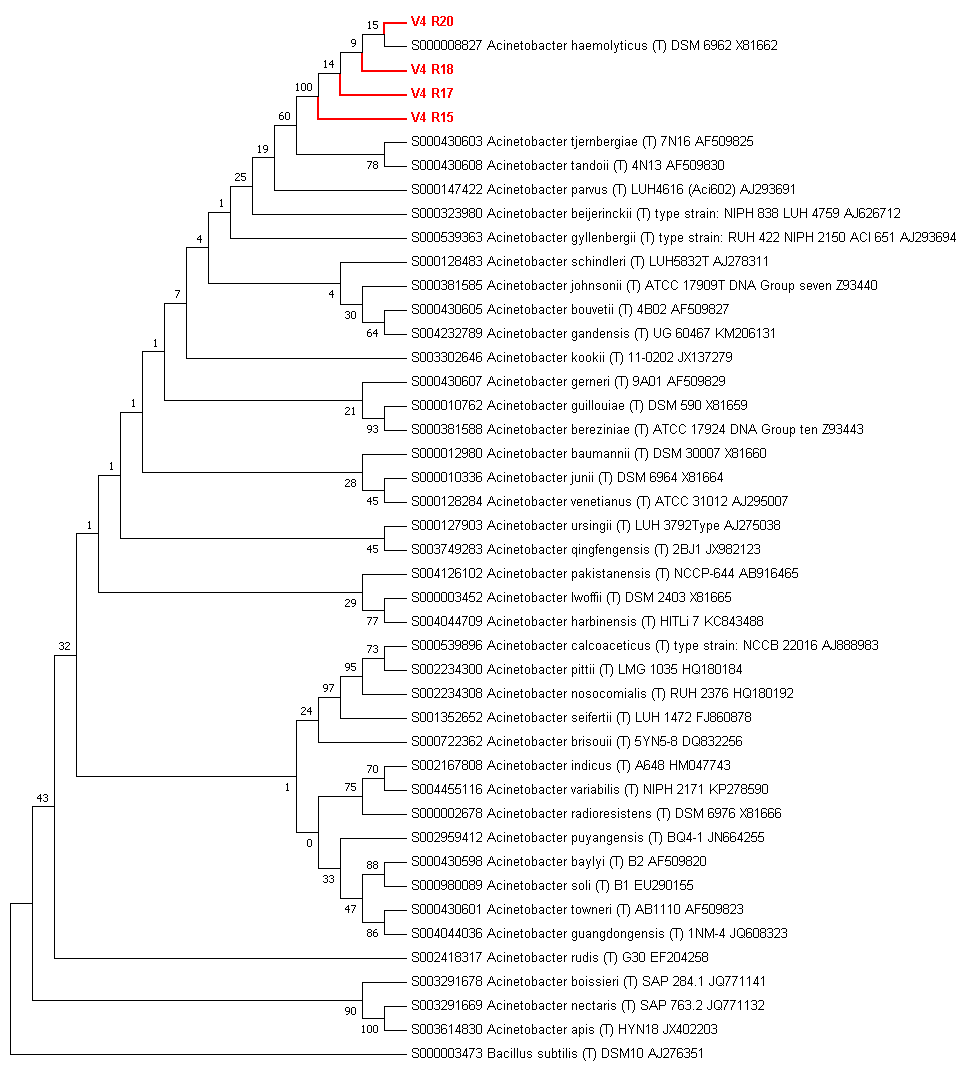


Figure S1: Phylogenetic tree of *Acinetobacter* isolates. Branches are color-coded according to the sampling of origin: first, olive; second, red; third, fuchsia, fourth, lime; fifth, blue.


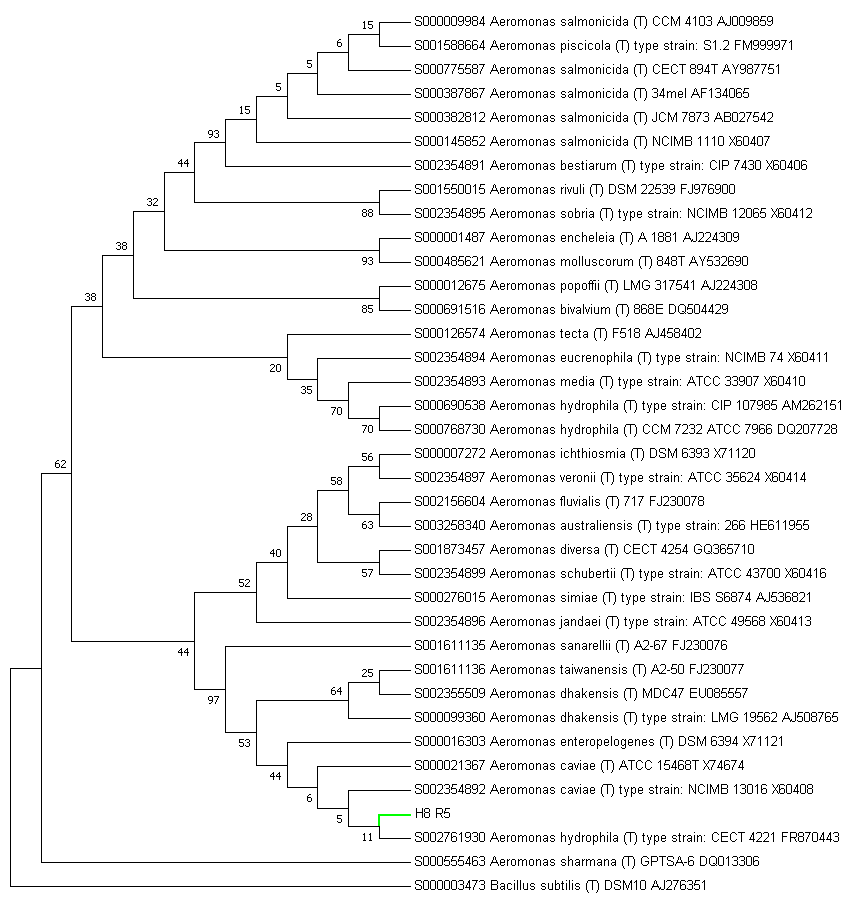


Figure S2: Phylogenetic tree of *Aeromonas* isolates. Branches are color-coded according to the sampling of origin: first, olive; second, red; third, fuchsia, fourth, lime; fifth, blue.


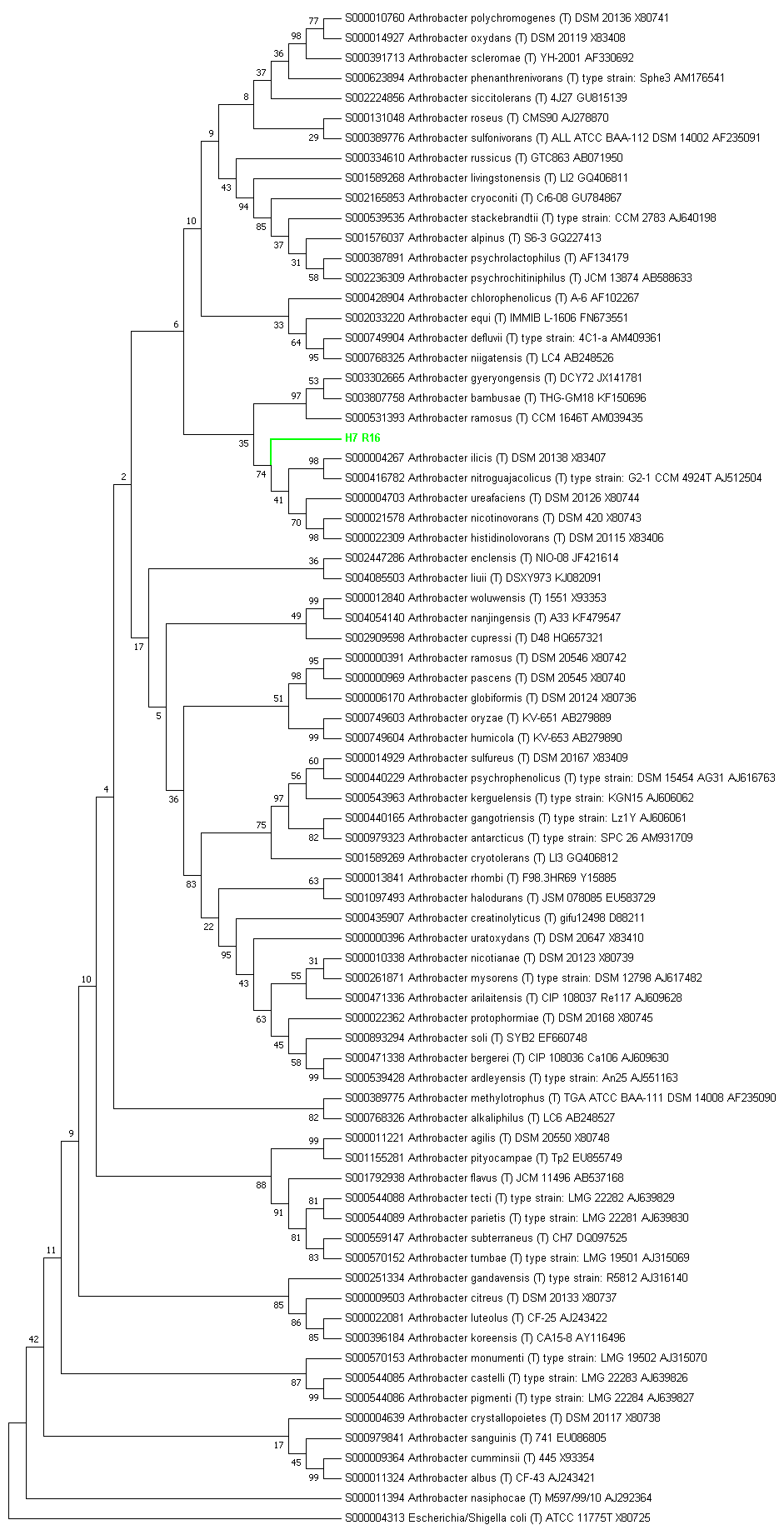


Figure S3: Phylogenetic tree of *Arthobacter* isolates. Branches are color-coded according to the sampling of origin: first, olive; second, red; third, fuchsia, fourth, lime; fifth, blue.


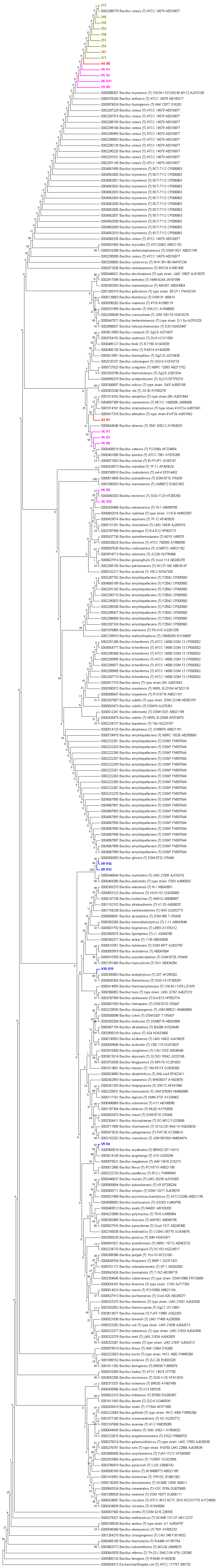


Figure S4: Phylogenetic tree of *Bacillus* isolates. Branches are color-coded according to the sampling of origin: first, olive; second, red; third, fuchsia, fourth, lime; fifth, blue.


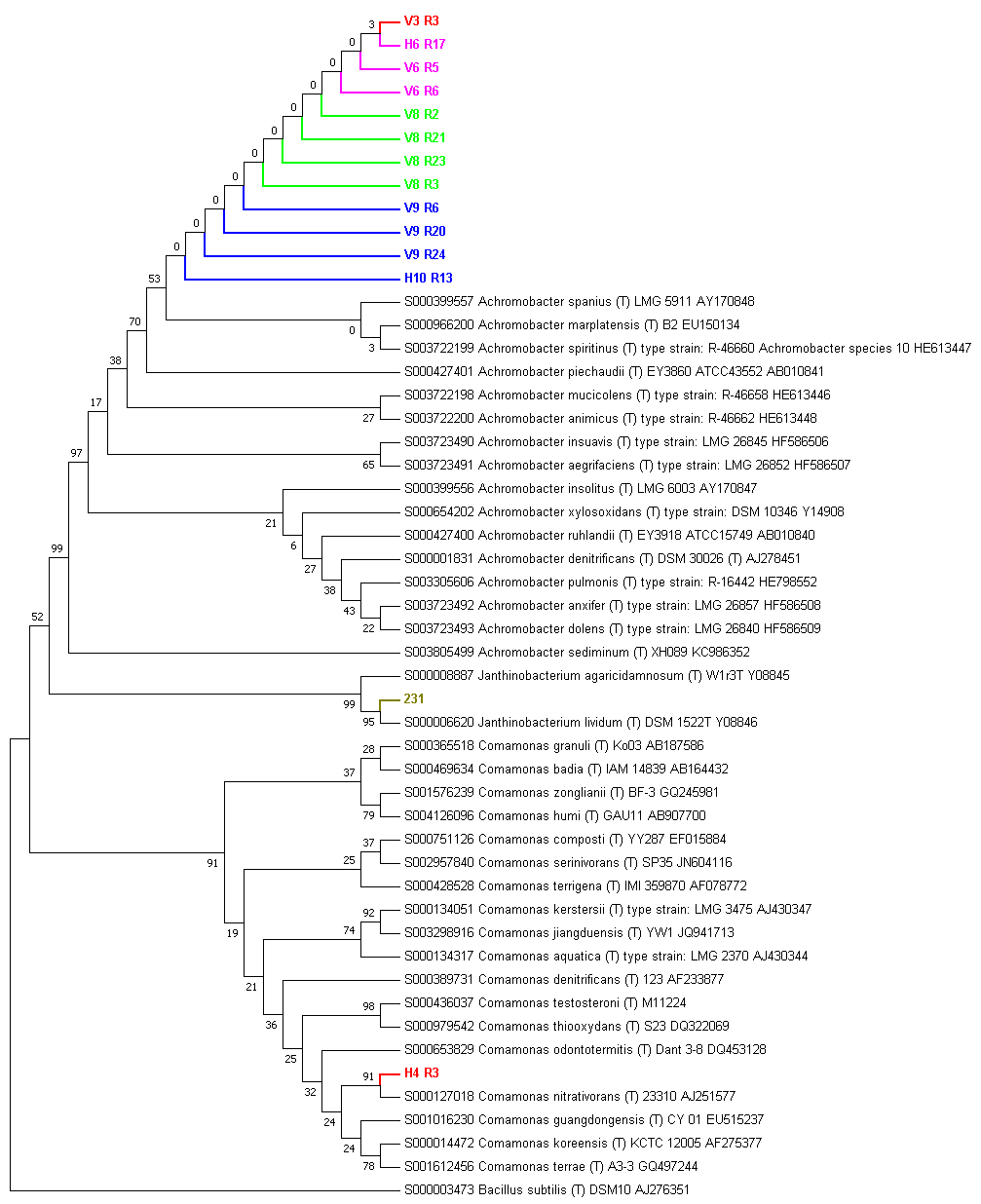


Figure S5: Phylogenetic tree of *Bhurkholderiales* isolates. Branches are color-coded according to the sampling of origin: first, olive; second, red; third, fuchsia, fourth, lime; fifth, blue.


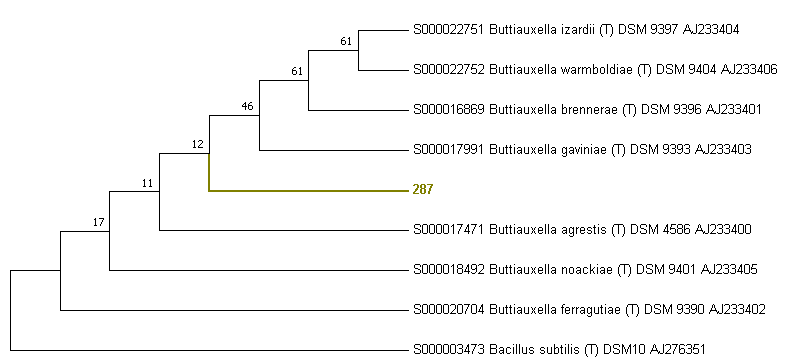


Figure S6: Phylogenetic tree of *Buttiauxella* isolates. Branches are color-coded according to the sampling of origin: first, olive; second, red; third, fuchsia, fourth, lime; fifth, blue.


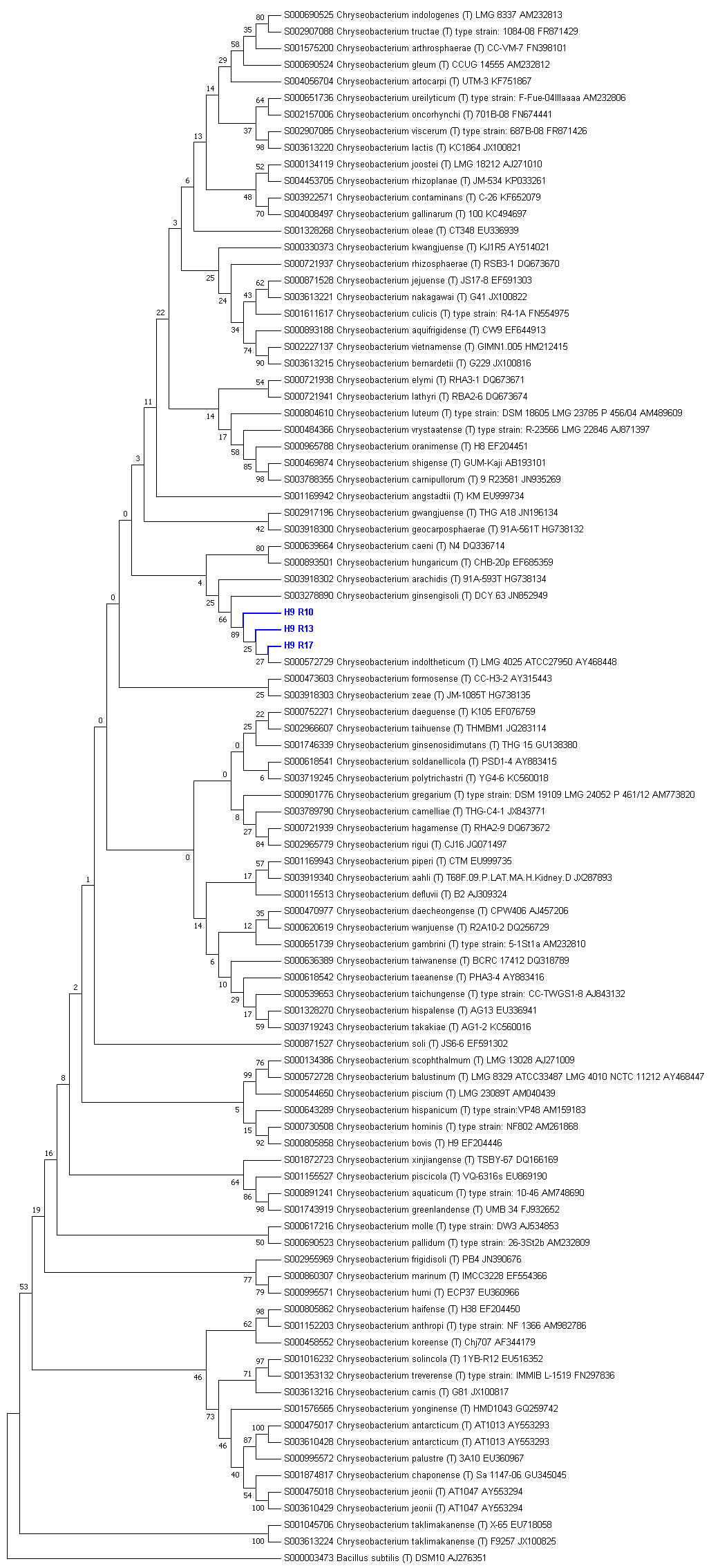


Figure S7: Phylogenetic tree of *Chryseobacterium* isolates. Branches are color-coded according to the sampling of origin: first, olive; second, red; third, fuchsia, fourth, lime; fifth, blue.


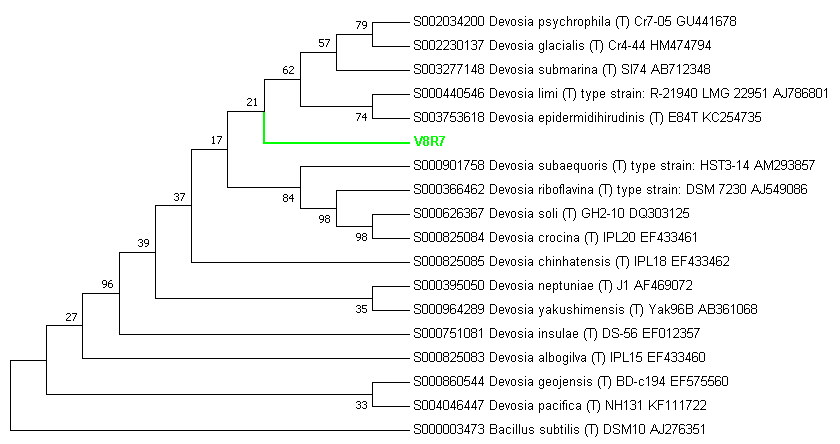


Figure S8: Phylogenetic tree of *Devosia* isolates. Branches are color-coded according to the sampling of origin: first, olive; second, red; third, fuchsia, fourth, lime; fifth, blue.


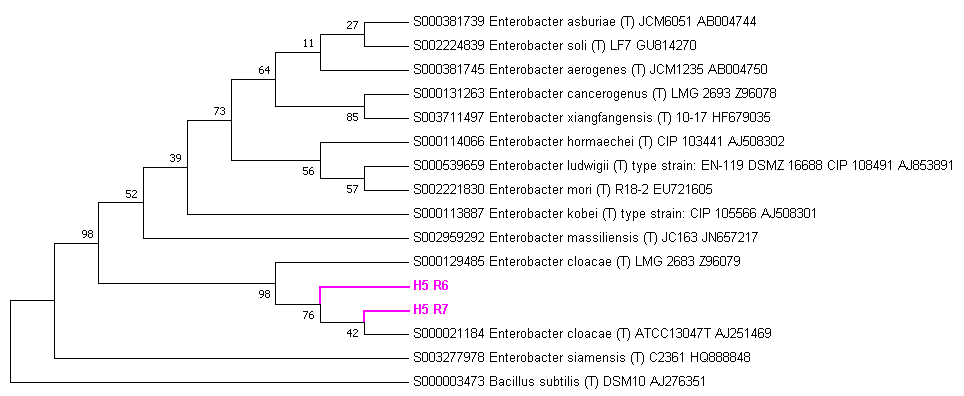


Figure S9: Phylogenetic tree of *Enterobacter* isolates. Branches are color-coded according to the sampling of origin: first, olive; second, red; third, fuchsia, fourth, lime; fifth, blue.


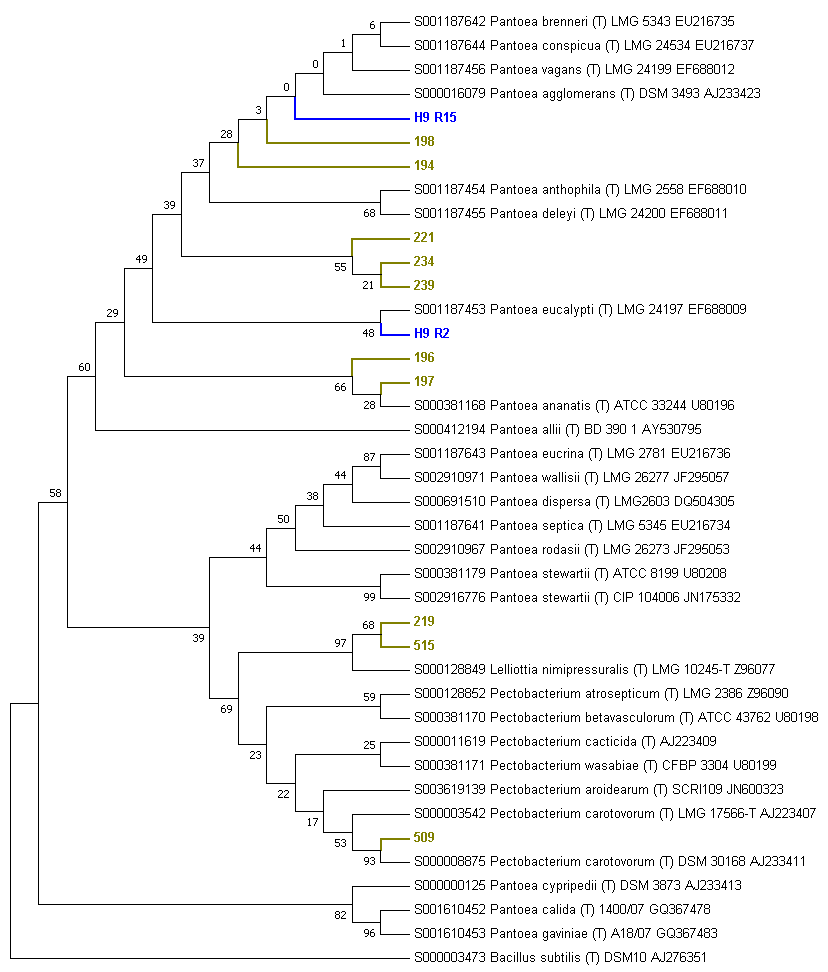


Figure S10: Phylogenetic tree of *Enterobacteriales* isolates. Branches are color-coded according to the sampling of origin: first, olive; second, red; third, fuchsia, fourth, lime; fifth, blue.


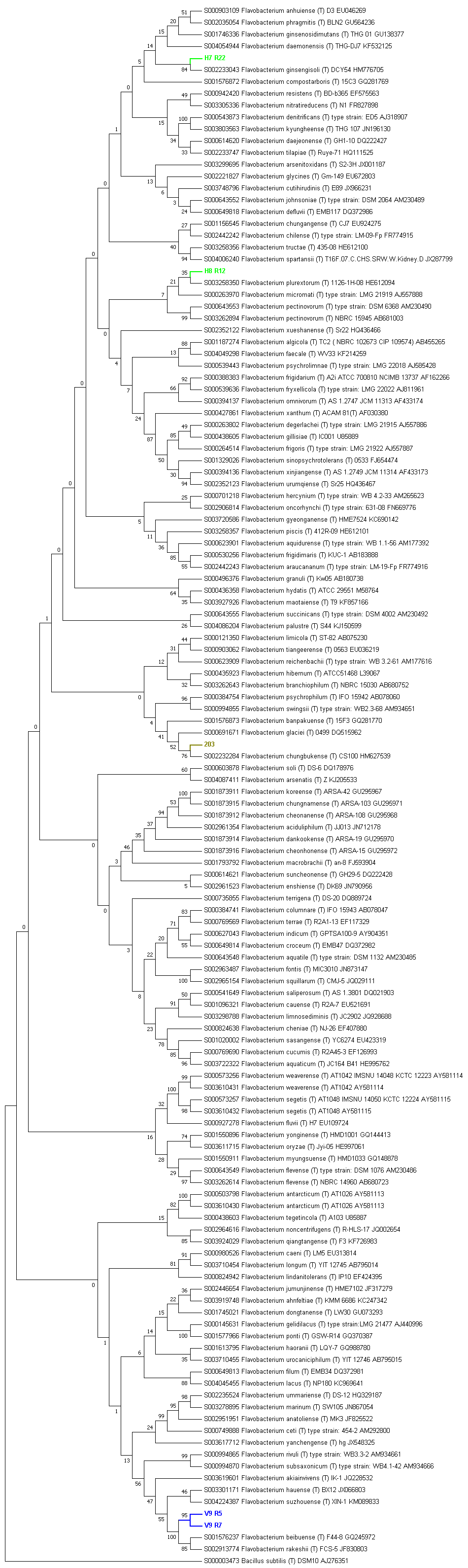


Figure S11: Phylogenetic tree of *Flavobacterium* isolates. Branches are color-coded according to the sampling of origin: first, olive; second, red; third, fuchsia, fourth, lime; fifth, blue.


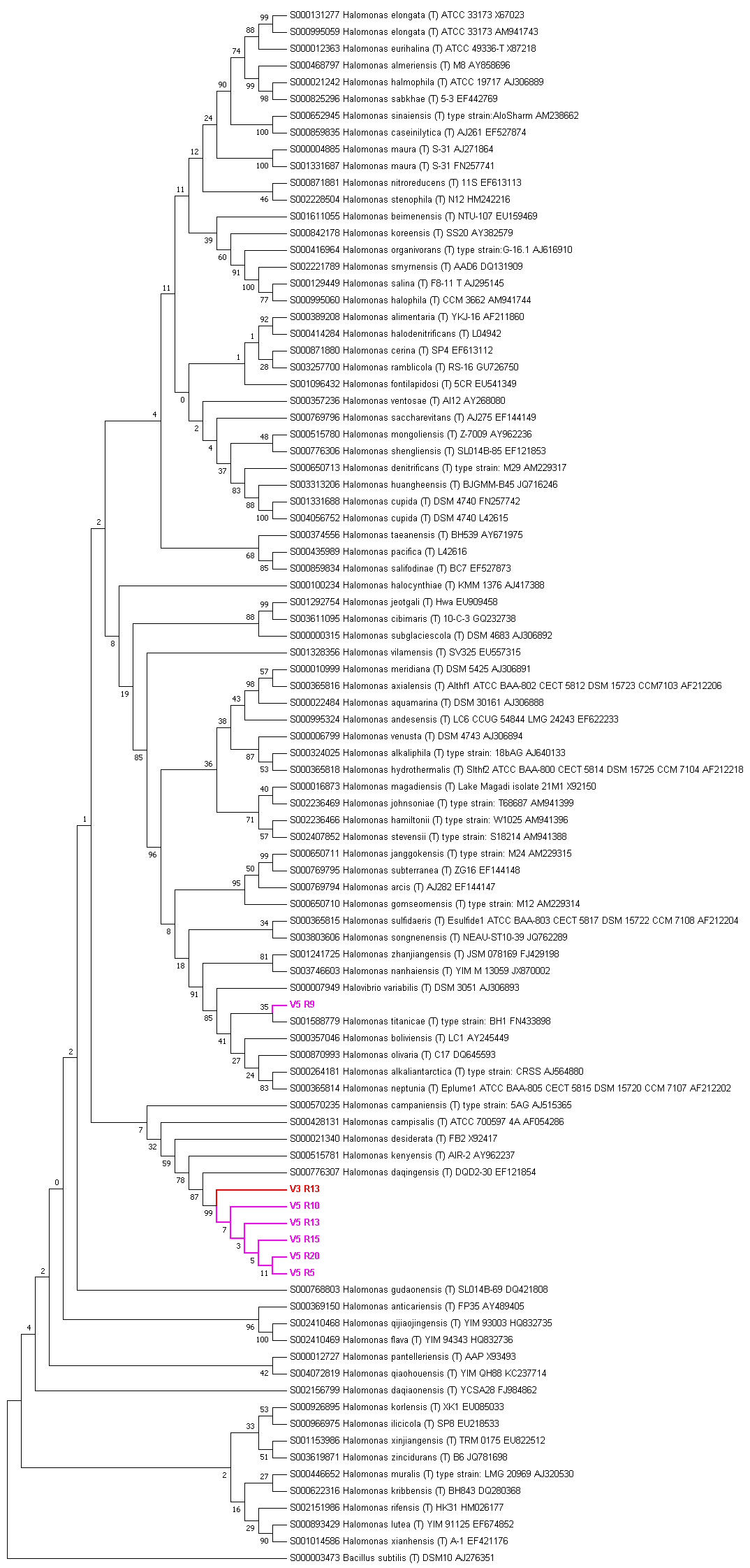


Figure S12: Phylogenetic tree of *Halomonas* isolates. Branches are color-coded according to the sampling of origin: first, olive; second, red; third, fuchsia, fourth, lime; fifth, blue.


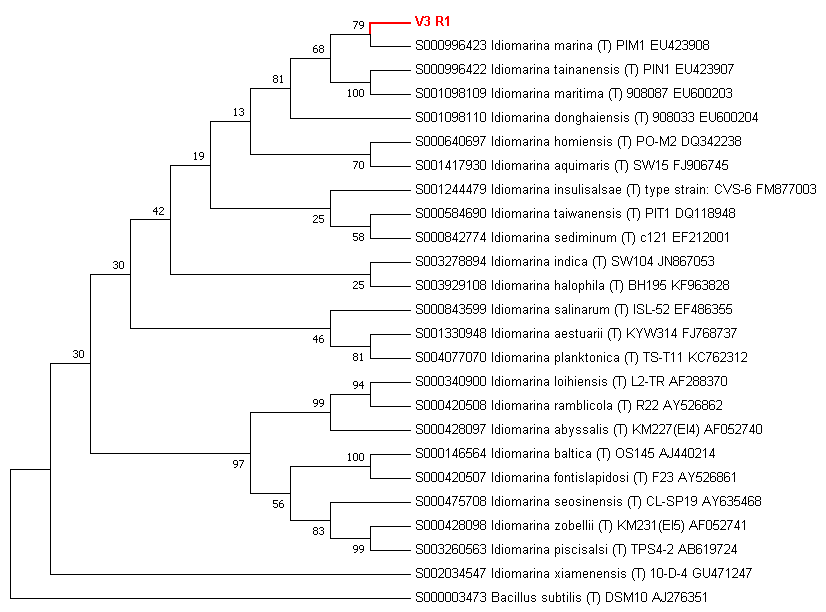


Figure S13: Phylogenetic tree of *Idiomarina* isolates. Branches are color-coded according to the sampling of origin: first, olive; second, red; third, fuchsia, fourth, lime; fifth, blue.


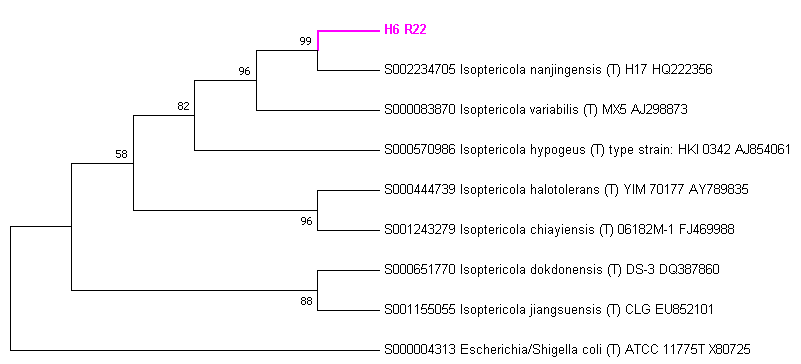


Figure S14: Phylogenetic tree of *Isoptericola* isolates. Branches are color-coded according to the sampling of origin: first, olive; second, red; third, fuchsia, fourth, lime; fifth, blue.


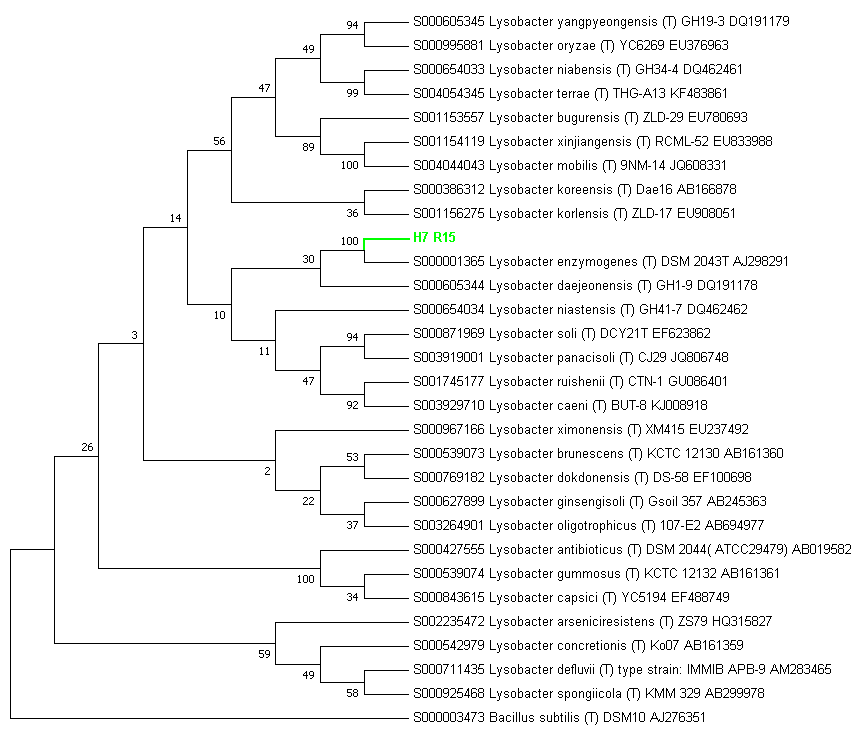


Figure S15: Phylogenetic tree of *Lysobacter* isolates. Branches are color-coded according to the sampling of origin: first, olive; second, red; third, fuchsia, fourth, lime; fifth, blue.


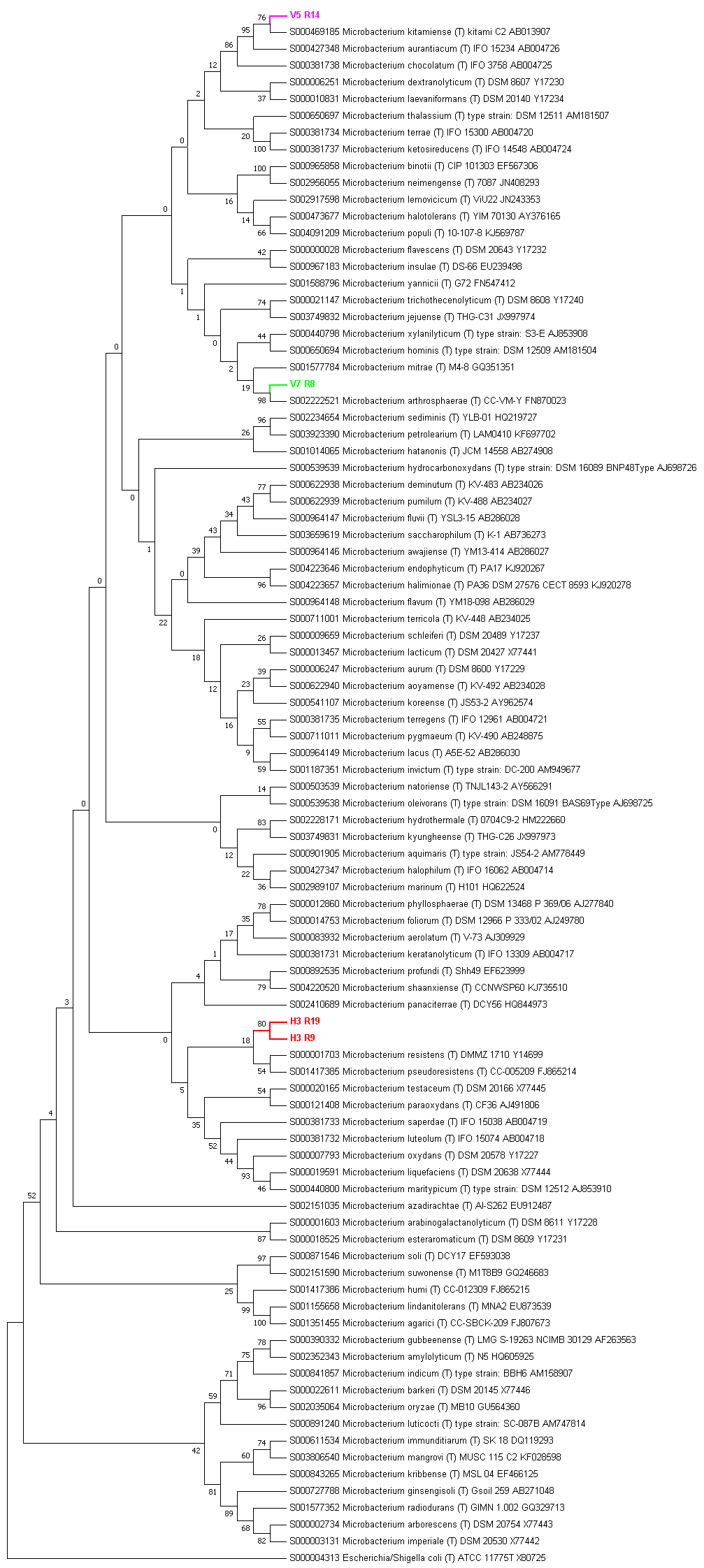


Figure S16: Phylogenetic tree of *Microbacterium* isolates. Branches are color-coded according to the sampling of origin: first, olive; second, red; third, fuchsia, fourth, lime; fifth, blue.


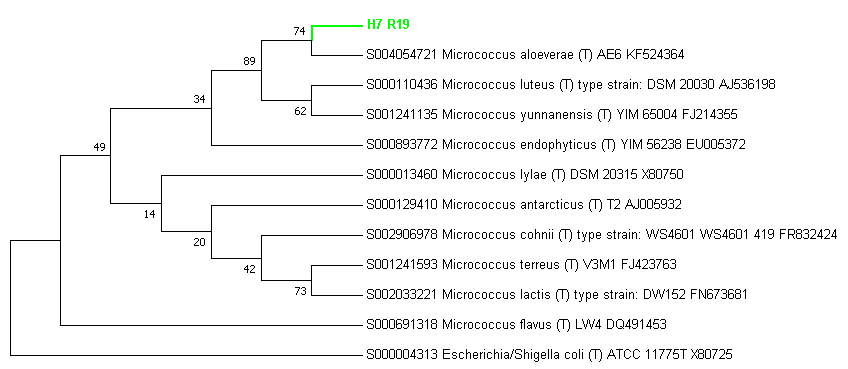


Figure S17: Phylogenetic tree of *Micrococcus* isolates. Branches are color-coded according to the sampling of origin: first, olive; second, red; third, fuchsia, fourth, lime; fifth, blue.


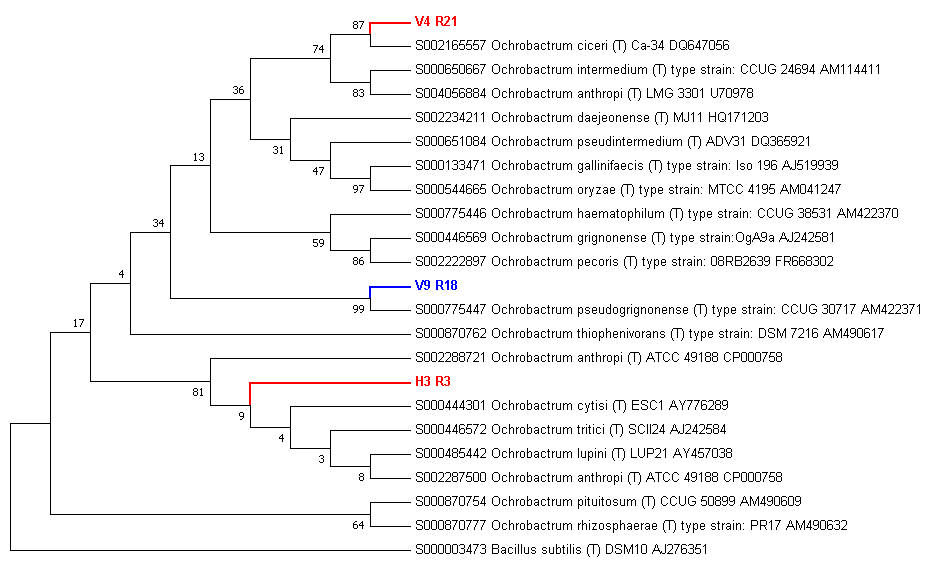


Figure S18: Phylogenetic tree of *Ochrobactrum* isolates. Branches are color-coded according to the sampling of origin: first, olive; second, red; third, fuchsia, fourth, lime; fifth, blue.


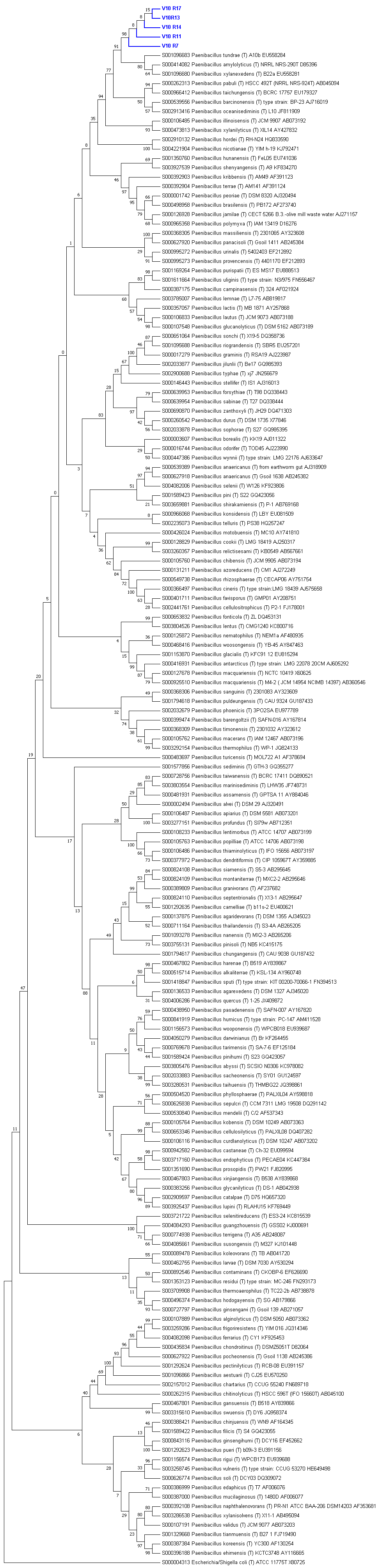


Figure S19: Phylogenetic tree of *Paenibacillus* isolates. Branches are color-coded according to the sampling of origin: first, olive; second, red; third, fuchsia, fourth, lime; fifth, blue.


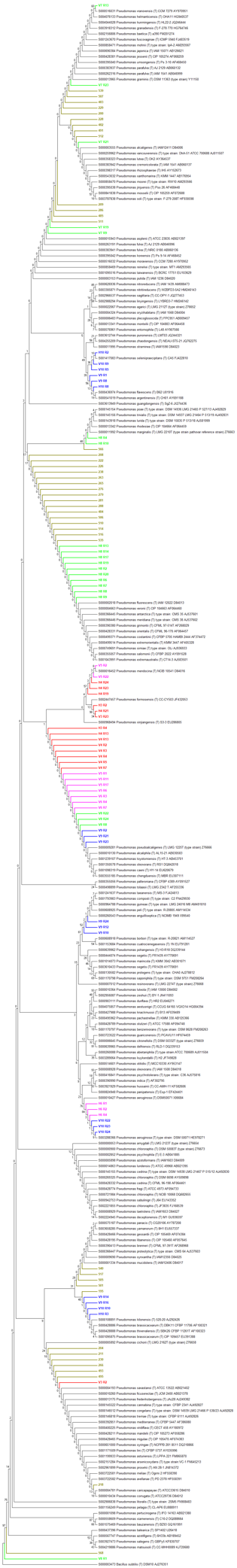


Figure S20: Phylogenetic tree of *Pseudomonas* isolates. Branches are color-coded according to the sampling of origin: first, olive; second, red; third, fuchsia, fourth, lime; fifth, blue.


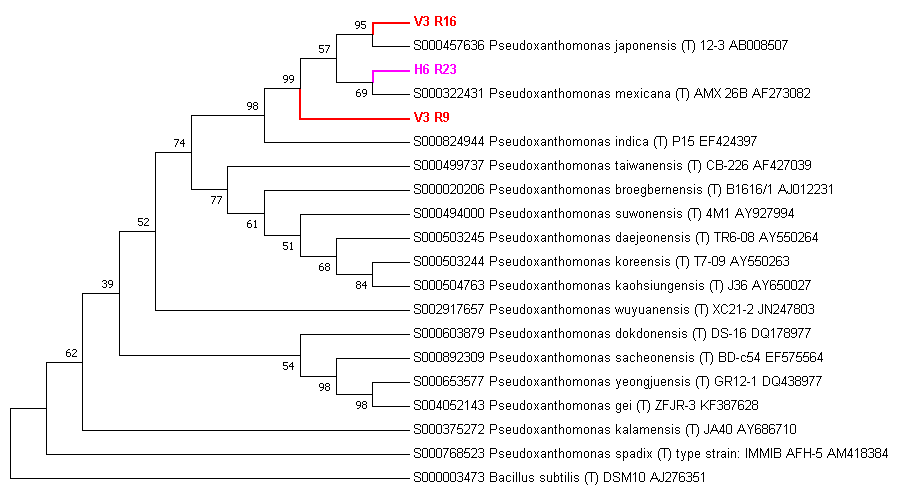


Figure S21: Phylogenetic tree of *Pseudoxanthomonas* isolates. Branches are color-coded according to the sampling of origin: first, olive; second, red; third, fuchsia, fourth, lime; fifth, blue.


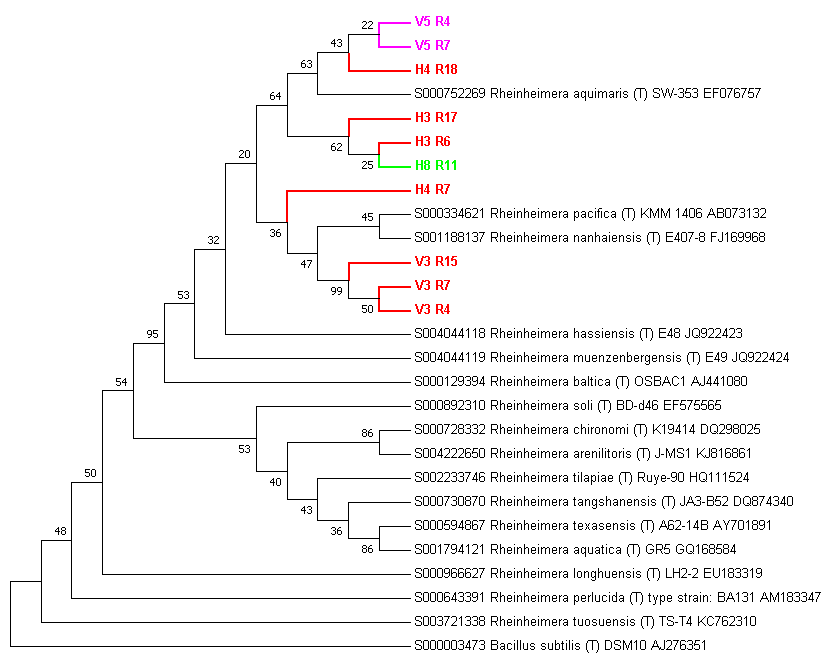


Figure S22: Phylogenetic tree of *Rheinheimera* isolates. Branches are color-coded according to the sampling of origin: first, olive; second, red; third, fuchsia, fourth, lime; fifth, blue.


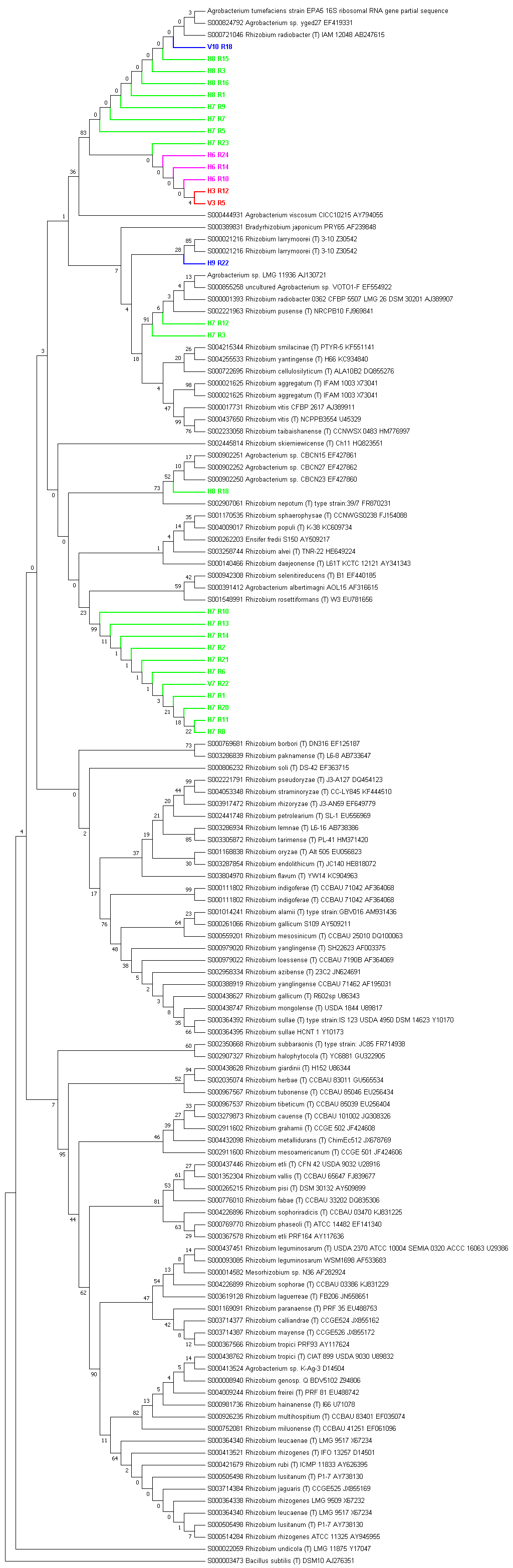


Figure S23: Phylogenetic tree of *Rhizobiales* isolates. Branches are color-coded according to the sampling of origin: first, olive; second, red; third, fuchsia, fourth, lime; fifth, blue.


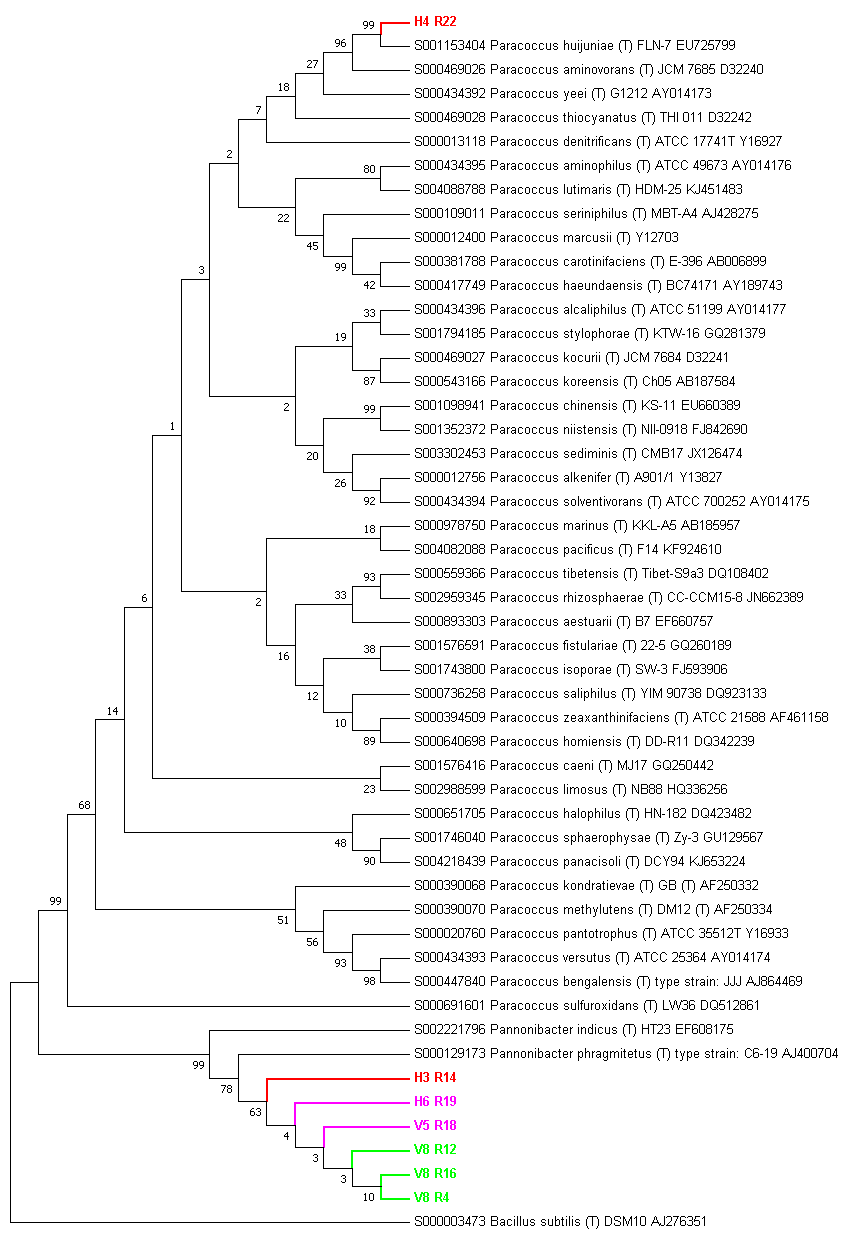


Figure S24: Phylogenetic tree of *Rhodobacterales* isolates. Branches are color-coded according to the sampling of origin: first, olive; second, red; third, fuchsia, fourth, lime; fifth, blue.


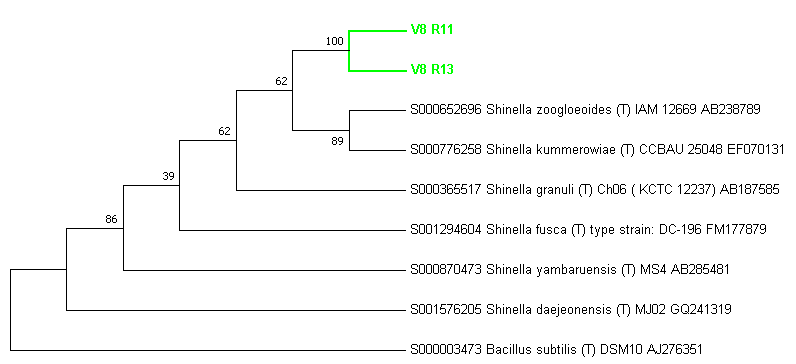


Figure S25: Phylogenetic tree of *Shinella* isolates. Branches are color-coded according to the sampling of origin: first, olive; second, red; third, fuchsia, fourth, lime; fifth, blue.


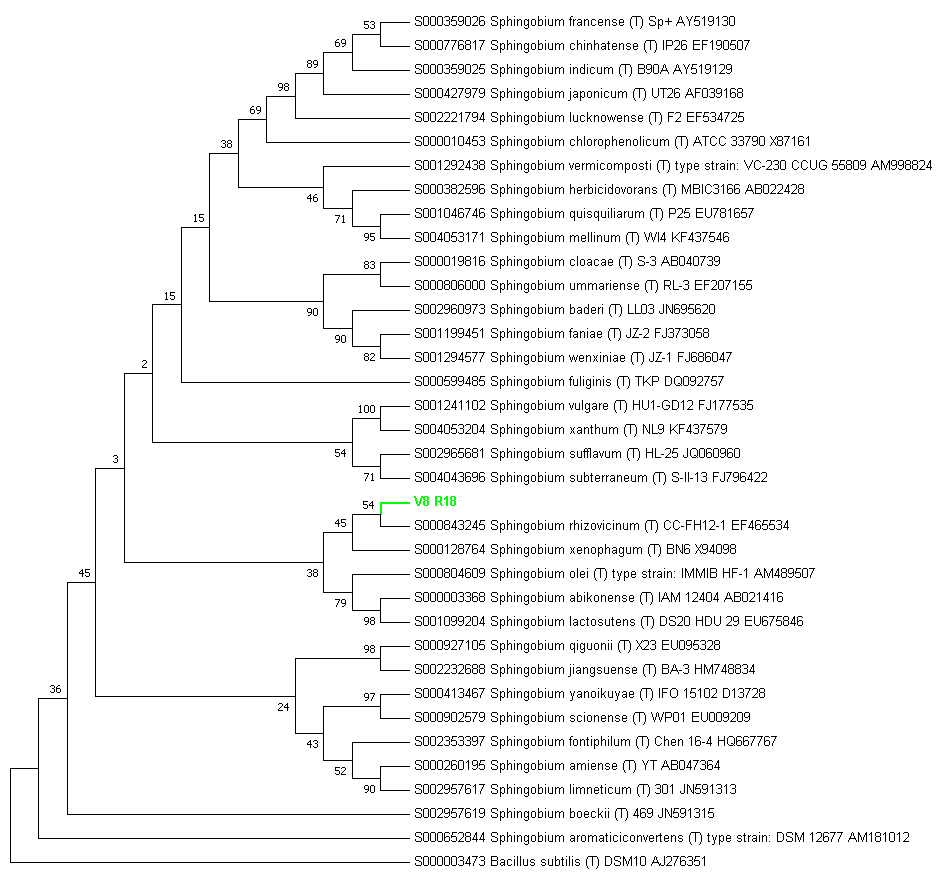


Figure S26: Phylogenetic tree of *Sphingobium* isolates. Branches are color-coded according to the sampling of origin: first, olive; second, red; third, fuchsia, fourth, lime; fifth, blue.


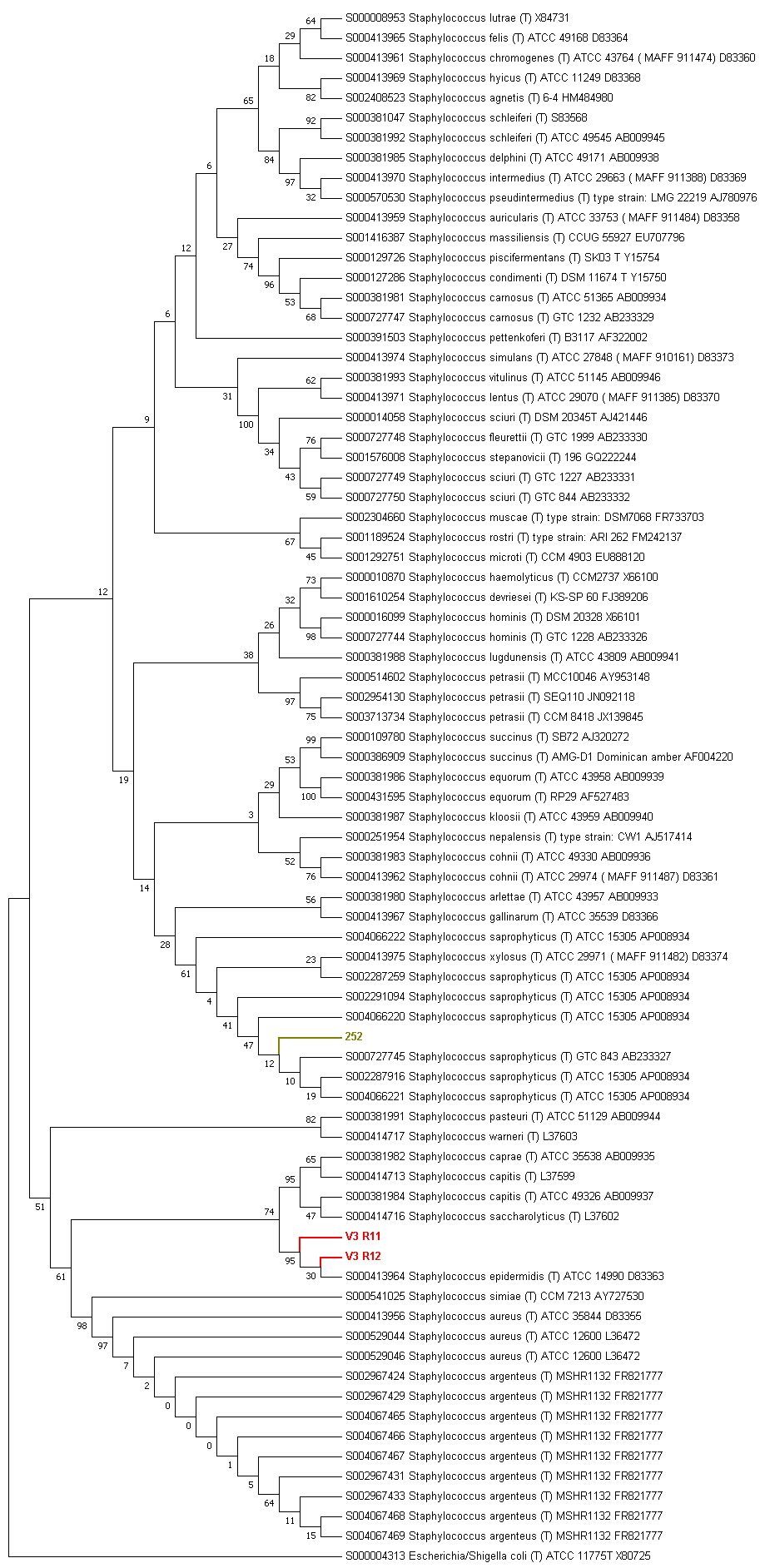


Figure S27: Phylogenetic tree of *Staphyloccoccus* isolates. Branches are color-coded according to the sampling of origin: first, olive; second, red; third, fuchsia, fourth, lime; fifth, blue.


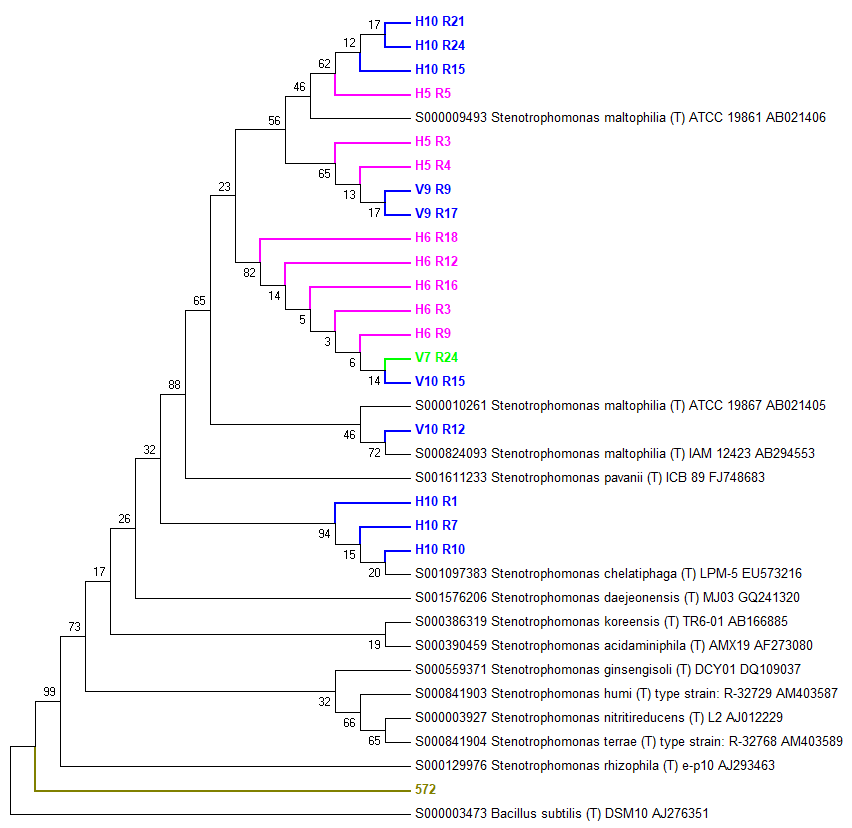


Figure S28: Phylogenetic tree of *Stenotrophomonas* isolates. Branches are color-coded according to the sampling of origin: first, olive; second, red; third, fuchsia, fourth, lime; fifth, blue.


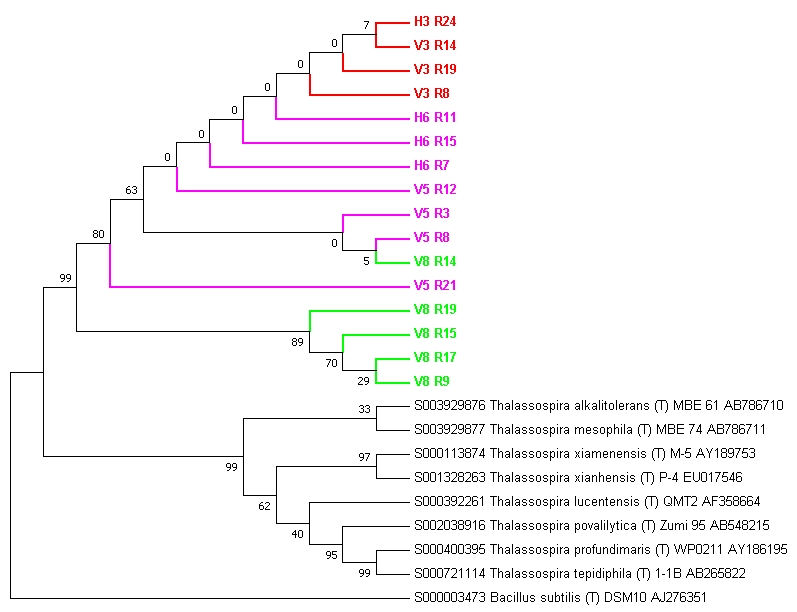


Figure S29: Phylogenetic tree of *Thalassospira* isolates. Branches are color-coded according to the sampling of origin: first, olive; second, red; third, fuchsia, fourth, lime; fifth, blue.


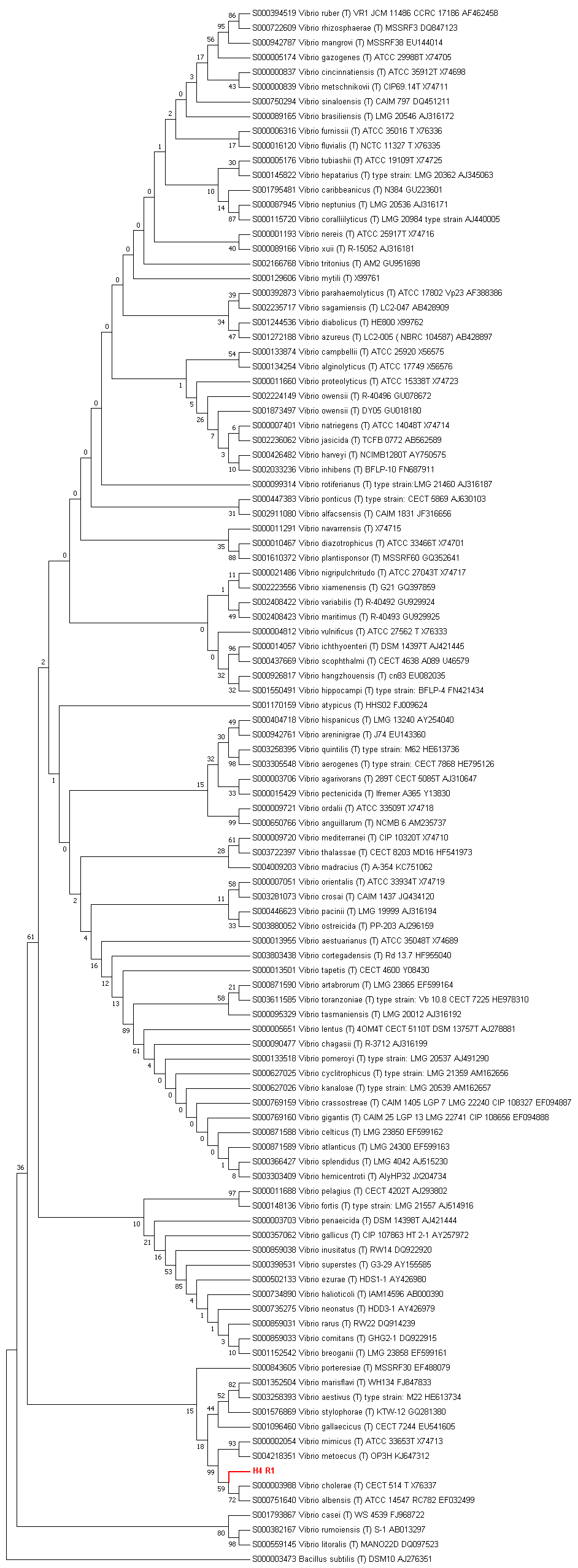


Figure S30: Phylogenetic tree of *Vibrio* isolates. Branches are color-coded according to the sampling of origin: first, olive; second, red; third, fuchsia, fourth, lime; fifth, blue.
