# Supplementary material for: Temporal Evolution of Bacterial Endophytes Associated to the Roots of Phragmites australis Exploited in Phytodepuration of Wastewater
Source: Front Microbiol. 2020 Jul 17;11:1652. doi: 10.3389/fmicb.2020.01652 (PMC7380131; doi:10.3389/fmicb.2020.01652)
Supplement: TABLE S1 — List of bacteria isolated from roots of Phragmites australis and used in this work. Accession numbers of 16S rRNA gene partial sequences are reported. [file Table_1.DOCX]

Table S1. List of bacteria isolated from roots of *Phragmites australis* and used in this work. Accession numbers of 16S rRNA gene partial sequences are reported.

| **Sampling** | **Isolate** | **Genus** | **Accession number** |
| --- | --- | --- | --- |
| 1 | V2 R1 | *Pantoea* | MK110895 |
| 1 | V2 R2 | *Pseudomonas* | MK110946 |
| 1 | V2 R3 | *Pantoea* | MK110896 |
| 1 | V2 R4 | *Pantoea* | MK110920 |
| 1 | V2 R5 | *Pantoea* | MK110921 |
| 1 | V2 R6 | *Pseudomonas* | MK110947 |
| 1 | V2 R7 | *Flavobacterium* | MK110948 |
| 1 | V2 R8 | *Pseudomonas* | MK110922 |
| 1 | V2 R9 | *Pseudomonas* | MK110897 |
| 1 | V2 R10 | *Pseudomonas* | MK110949 |
| 1 | V2 R12 | *Pseudomonas* | MK110898 |
| 1 | V2 R13 | *Pseudomonas* | MK110950 |
| 1 | V2 R14 | *Lelliottia* | MK110899 |
| 1 | V2 R15 | *Pantoea* | MK110959 |
| 1 | V2 R16 | *Pseudomonas* | MK110923 |
| 1 | V2 R17 | *Pseudomonas* | MK110925 |
| 1 | V2 R18 | *Pseudomonas* | MK110924 |
| 1 | V2 R19 | *Pseudomonas* | MK110960 |
| 1 | V2 R20 | *Pseudomonas* | MK110926 |
| 1 | V2 R21 | *Janthinobacterium* | MK110945 |
| 1 | V2 R22 | *Pantoea* | MK110957 |
| 1 | V2 R23 | *Pseudomonas* | MK110927 |
| 1 | V2 R24 | *Pantoea* | MK110928 |
| 1 | V1 R1 | *Bacillus* | MK110929 |
| 1 | V1 R2 | *Bacillus* | MK110930 |
| 1 | V1 R3 | *Staphylococcus* | MK110931 |
| 1 | V1 R4 | *Bacillus* | MK110932 |
| 1 | V1 R5 | *Bacillus* | MK110900 |
| 1 | V1 R6 | *Bacillus* | MK110901 |
| 1 | V1 R7 | *Pseudomonas* | MK110902 |
| 1 | V1 R8 | *Pseudomonas* | MK110933 |
| 1 | V1 R9 | *Pseudomonas* | MK110934 |
| 1 | V1 R10 | *Bacillus* | MK110935 |
| 1 | V1 R11 | *Bacillus* | MK110958 |
| 1 | V1 R12 | *Bacillus* | MK110936 |
| 1 | V1 R13 | *Bacillus* | MK110937 |
| 1 | V1 R15 | *Pseudomonas* | MK110938 |
| 1 | V1 R16 | *Pseudomonas* | MK110939 |
| 1 | V1 R17 | *Pseudomonas* | MK110940 |
| 1 | V1 R19 | *Pseudomonas* | MK110941 |
| 1 | V1 R20 | *Buttiauxella* | MK110942 |
| 1 | V1 R21 | *Pseudomonas* | MK110943 |
| 1 | H1 R2 | *Pseudomonas* | MK110903 |
| 1 | H1 R3 | *Pseudomonas* | MK110904 |
| 1 | H1 R4 | *Pseudomonas* | MK110905 |
| 1 | H1 R5 | *Pseudomonas* | MK110906 |
| 1 | H1 R6 | *Pseudomonas* | MK110907 |
| 1 | H1 R7 | *Pseudomonas* | MK110908 |
| 1 | H1 R9 | *Pseudomonas* | MK110909 |
| 1 | H1 R10 | *Pseudomonas* | MK110910 |
| 1 | H1 R13 | *Pseudomonas* | MK110911 |
| 1 | H1 R14 | *Pseudomonas* | MK110912 |
| 1 | H1 R15 | *Pseudomonas* | MK110951 |
| 1 | H1 R16 | *Pectobacterium* | MK110913 |
| 1 | H1 R17 | *Pseudomonas* | MK110914 |
| 1 | H1 R18 | *Pseudomonas* | MK110915 |
| 1 | H1 R19 | *Pseudomonas* | MK110952 |
| 1 | H1 R20 | *Pseudomonas* | MK110916 |
| 1 | H1 R21 | *Lelliottia* | MK110953 |
| 1 | H1 R22 | *Pseudomonas* | MK110917 |
| 1 | H1 R23 | *Pseudomonas* | MK110954 |
| 1 | H2 R1 | *Pseudomonas* | MK110918 |
| 1 | H2 R2 | *Pseudomonas* | MK110944 |
| 1 | H2 R3 | *Pseudomonas* | MK110961 |
| 1 | H2 R5 | *Pseudomonas* | MK110919 |
| 1 | H2 R6 | *Pseudomonas* | MK110955 |
| 1 | H2 R7 | *Stenotrophomonas* | MK110956 |

| 2 | V3 R3 | *Achromobacter* | | MK134509 | |
| --- | --- | --- | --- | --- | --- |
| 2 | V4 R15 | *Acinetobacter* | | MK134489 | |
| 2 | V4 R17 | *Acinetobacter* | | MK134488 | |
| 2 | V4 R18 | *Acinetobacter* | | MK134487 | |
| 2 | V4 R20 | *Acinetobacter* | | MK134486 | |
| 2 | H3 R12 | *Agrobacterium* | | MK134554 | |
| 2 | V3 R5 | *Agrobacterium* | | MK134508 | |
| 2 | H4 R8 | *Bacillus* | | MK134547 | |
| 2 | V4 R1 | *Bacillus* | | MK134496 | |
| 2 | H4 R3 | *Comamonas* | | MK138850 | |
| 2 | V3 R13 | *Halomonas* | | MK134502 | |
| 2 | V3 R1 | *Idiomarina* | | MK134511 | |
| 2 | H3 R19 | *Microbacterium* | | MK134551 | |
| 2 | H3 R9 | *Microbacterium* | | MK134555 | |
| 2 | H3 R2 | *Pseudomonas* | | MK134559 | |
| 2 | H3 R3 | *Ochrobactrum* | | MK134558 | |
| 2 | H4 R1 | *Vibrio* | | MK134549 | |
| 2 | V4 R21 | *Ochrobactrum* | | MK138851 | |
| 2 | H3 R14 | *Pannonibacter* | | MK134553 | |
| 2 | H4 R22 | *Paracoccus* | | MK134542 | |
| 2 | H3 R4 | *Pseudomonas* | | MK134557 | |
| 2 | H4 R13 | *Pseudomonas* | | MK134546 | |
| 2 | H4 R19 | *Pseudomonas* | | MK134544 | |
| 2 | H4 R21 | *Pseudomonas* | | MK134543 | |
| 2 | H4 R23 | *Pseudomonas* | | MK134541 | |
| 2 | H4 R24 | *Pseudomonas* | | MK134540 | |
| 2 | V3 R2 | *Pseudomonas* | | MK134510 | |
| 2 | V3 R23 | *Pseudomonas* | | MK134497 | |
| 2 | V4 R13 | *Pseudomonas* | | MK134490 | |
| 2 | V4 R2 | *Pseudomonas* | | MK134495 | |
| 2 | V4 R3 | *Pseudomonas* | | MK134494 | |
| 2 | V4 R4 | *Pseudomonas* | | MK134493 | |
| 2 | V3 R16 | *Pseudoxanthomonas* | | MK134499 | |
| 2 | V3 R9 | *Pseudoxanthomonas* | | MK134505 | |
| 2 | H3 R17 | *Rheinheimera* | | MK134552 | |
| 2 | H3 R6 | *Rheinheimera* | | MK134556 | |
| 2 | H4 R18 | *Rheinheimera* | | MK134545 | |
| 2 | H4 R7 | *Rheinheimera* | | MK134548 | |
| 2 | V3 R15 | *Rheinheimera* | | MK134500 | |
| 2 | V3 R4 | *Rheinheimera* | | MT165525 | |
| 2 | V3 R7 | *Rheinheimera* | | MK134507 | |
| 2 | V3 R11 | *Staphylococcus* | | MK134504 | |
| 2 | V3 R12 | *Staphylococcus* | | MK134503 | |
| 2 | H3 R24 | *Thalassospira* | | MK134550 | |
| 2 | V3 R14 | *Thalassospira* | | MK134501 | |
| 2 | V3 R19 | *Thalassospira* | | MK134498 | |
| 2 | V3 R8 | *Thalassospira* | | MK134506 | |
| 2 | V4 R5 | *Pseudomonas* | | MK134492 | |
| 2 | V4 R7 | *Pseudomonas* | | MK134491 | |
| 3 | H6 R17 | *Achromobacter* | | MK134518 | |
| 3 | V6 R5 | *Achromobacter* | | MK130934 | |
| 3 | V6 R6 | *Achromobacter* | | MK130935 | |
| 3 | H6 R10 | *Agrobacterium* | | MK134524 | |
| 3 | H5 R1 | *Bacillus* | | MK134539 | |
| 3 | H5 R2 | *Bacillus* | | MK134538 | |
| 3 | H6 R20 | *Bacillus* | | MK134515 | |
| 3 | H6 R21 | *Bacillus* | | MK138852 | |
| 3 | H6 R8 | *Bacillus* | | MK134526 | |
| 3 | V6 R1 | *Bacillus* | | MK130907 | |
| 3 | V6 R2 | *Bacillus* | | MK130906 | |
| 3 | V6 R8 | *Bacillus* | | MK130937 | |
| 3 | H5 R6 | *Enterobacter* | | MK134534 | |
| 3 | H5 R7 | *Enterobacter* | | MK134533 | |
| 3 | V5 R10 | *Halomonas* | | MK138853 | |
| 3 | V5 R13 | *Halomonas* | | MK130915 | |
| 3 | V5 R15 | *Halomonas* | | MK130913 | |
| 3 | V5 R20 | *Halomonas* | | MK130910 | |
| 3 | V5 R5 | *Halomonas* | | MK130921 | |
| 3 | V5 R9 | *Halomonas* | | MK130917 | |
| 3 | H6 R22 | *Isoptericola* | | MK134514 | |
| 3 | V5 R14 | *Microbacterium* | | MK130914 | |
| 3 | H6 R19 | *Pannonibacter* | | MK134516 | |
| 3 | V5 R18 | *Pannonibacter* | | MK130911 | |
| 3 | H5 R8 | *Bacillus* | | MK134532 | |
| 3 | H6 R1 | *Pseudomonas* | | MK134531 | |
| 3 | H6 R2 | *Pseudomonas* | | MK134530 | |
| 3 | H6 R4 | *Pseudomonas* | | MK134528 | |
| 3 | V5 R1 | *Pseudomonas* | | MK134485 | |
| 3 | V5 R11 | *Pseudomonas* | | MK138854 | |
| 3 | V5 R17 | *Pseudomonas* | | MK130912 | |
| 3 | V5 R22 | *Pseudomonas* | | MK130908 | |
| 3 | V5 R6 | *Pseudomonas* | | MK130920 | |
| 3 | V6 R3 | *Pseudomonas* | | MK130932 | |
| 3 | V6 R4 | *Pseudomonas* | | MK130933 | |
| 3 | V6 R7 | *Pseudomonas* | | MK130936 | |
| 3 | H6 R23 | *Pseudoxanthomonas* | | MK134513 | |
| 3 | V5 R4 | *Rheinheimera* | | MK130922 | |
| 3 | V5 R7 | *Rheinheimera* | | MK130919 | |
| 3 | H6 R14 | *Rhizobium* | | MK134521 | |
| 3 | H6 R24 | *Rhizobium* | | MK134512 | |
| 3 | H5 R3 | *Stenotrophomonas* | | MK134537 | |
| 3 | H5 R4 | *Stenotrophomonas* | | MK134536 | |
| 3 | H5 R5 | *Stenotrophomonas* | | MK134535 | |
| 3 | H6 R12 | *Stenotrophomonas* | | MK134522 | |
| 3 | H6 R16 | *Stenotrophomonas* | | MK134519 | |
| 3 | H6 R18 | *Stenotrophomonas* | | MK134517 | |
| 3 | H6 R3 | *Stenotrophomonas* | | MK134529 | |
| 3 | H6 R9 | *Stenotrophomonas* | | MK134525 | |
| 3 | H6 R11 | *Thalassospira* | | MK134523 | |
| 3 | H6 R15 | *Thalassospira* | | MK134520 | |
| 3 | H6 R7 | *Thalassospira* | | MK134527 | |
| 3 | V5 R12 | *Thalassospira* | | MK130916 | |
| 3 | V5 R2 | *Pseudomonas* | | MK130931 | |
| 3 | V5 R21 | *Thalassospira* | | MK130909 | |
| 3 | V5 R3 | *Thalassospira* | | MK130923 | |
| 3 | V5 R8 | *Thalassospira* | | MK130918 | |
| 4 | V8 R2 | | *Achromobacter* | MK130945 |  |
| 4 | V8 R21 | | *Achromobacter* | MK130957 |  |
| 4 | V8 R23 | | *Achromobacter* | MK130901 |  |
| 4 | V8 R3 | | *Achromobacter* | MK130905 |  |
| 4 | H8 R5 | | *Aeromonas* | MK133358 |  |
| 4 | H7 R23 | | *Agrobacterium* | MK138868 |  |
| 4 | H7 R3 | | *Agrobacterium* | MK138869 |  |
| 4 | H7 R5 | | *Agrobacterium* | MK138870 |  |
| 4 | H7 R7 | | *Agrobacterium* | MK138872 |  |
| 4 | H7 R9 | | *Agrobacterium* | MK138874 |  |
| 4 | H8 R1 | | *Agrobacterium* | MK138875 |  |
| 4 | H8 R16 | | *Agrobacterium* | MK130924 |  |
| 4 | H8 R18 | | *Agrobacterium* | MK130928 |  |
| 4 | H8 R3 | | *Agrobacterium* | MK138881 |  |
| 4 | H7 R16 | | *Arthrobacter* | MK138862 |  |
| 4 | V8R7 | | *Devosia* | MK130903 |  |
| 4 | H7 R22 | | *Flavobacterium* | MK138867 |  |
| 4 | H8 R12 | | *Flavobacterium* | MK138878 |  |
| 4 | H7 R15 | | *Lysobacter* | MK138861 |  |
| 4 | V7 R8 | | *Microbacterium* | MK130939 |  |
| 4 | H7 R19 | | *Micrococcus* | MK138863 |  |
| 4 | V8 R12 | | *Pannonibacter* | MK130949 |  |
| 4 | V8 R16 | | *Pannonibacter* | MK130953 |  |
| 4 | V8 R4 | | *Pannonibacter* | MK130904 |  |
| 4 | H8 R10 | | *Pseudomonas* | MK138876 |  |
| 4 | H8 R13 | | *Pseudomonas* | MK138879 |  |
| 4 | H8 R14 | | *Pseudomonas* | MK130926 |  |
| 4 | H8 R17 | | *Pseudomonas* | MK130927 |  |
| 4 | H8 R19 | | *Pseudomonas* | MK130929 |  |
| 4 | H8 R2 | | *Pseudomonas* | MK138880 |  |
| 4 | H8 R20 | | *Pseudomonas* | MK130930 |  |
| 4 | H8 R4 | | *Pseudomonas* | MK138882 |  |
| 4 | H8 R6 | | *Pseudomonas* | MK138883 |  |
| 4 | H8 R7 | | *Pseudomonas* | MK138884 |  |
| 4 | H8 R8 | | *Pseudomonas* | MK138885 |  |
| 4 | H8 R9 | | *Pseudomonas* | MK138886 |  |
| 4 | V7 R13 | | *Pseudomonas* | MK138887 |  |
| 4 | V7 R19 | | *Pseudomonas* | MK130940 |  |
| 4 | V7 R21 | | *Pseudomonas* | MK130941 |  |
| 4 | V7 R23 | | *Pseudomonas* | MK130943 |  |
| 4 | V7 R9 | | *Pseudomonas* | MK138889 |  |
| 4 | V8 R1 | | *Pseudomonas* | MK130944 |  |
| 4 | V8 R22 | | *Pseudomonas* | MK130902 |  |
| 4 | V8 R24 | | *Pseudomonas* | MK130900 |  |
| 4 | V8 R8 | | *Pseudomonas* | MK130946 |  |
| 4 | H8 R11 | | *Rheinheimera* | MK138877 |  |
| 4 | H7 R1 | | *Rhizobium* | MK138855 |  |
| 4 | H7 R10 | | *Rhizobium* | MK138856 |  |
| 4 | H7 R11 | | *Rhizobium* | MK138857 |  |
| 4 | H7 R12 | | *Rhizobium* | MK138858 |  |
| 4 | H7 R13 | | *Rhizobium* | MK138859 |  |
| 4 | H7 R14 | | *Rhizobium* | MK138860 |  |
| 4 | H7 R2 | | *Rhizobium* | MK138864 |  |
| 4 | H7 R20 | | *Rhizobium* | MK138865 |  |
| 4 | H7 R21 | | *Rhizobium* | MK138866 |  |
| 4 | H7 R6 | | *Rhizobium* | MK138871 |  |
| 4 | H7 R8 | | *Rhizobium* | MK138873 |  |
| 4 | H8 R15 | | *Rhizobium* | MK130925 |  |
| 4 | V7 R22 | | *Rhizobium* | MK130942 |  |
| 4 | V8 R11 | | *Shinella* | MK130948 |  |
| 4 | V8 R13 | | *Shinella* | MK130950 |  |
| 4 | V8 R18 | | *Sphingobium* | MK130955 |  |
| 4 | V7 R24 | | *Stenotrophomonas* | MK138888 |  |
| 4 | V8 R14 | | *Thalassospira* | MK130951 |  |
| 4 | V8 R15 | | *Thalassospira* | MK130952 |  |
| 4 | V8 R17 | | *Thalassospira* | MK130954 |  |
| 4 | V8 R19 | | *Thalassospira* | MK130956 |  |
| 4 | V8 R9 | | *Thalassospira* | MK130947 |  |

| 5 | V9 R1 | *Pseudomonas* | MT165526 |
| --- | --- | --- | --- |
| 5 | V9 R2 | *Pseudomonas* | MT165527 |
| 5 | V9 R4 | *Bacillus* | MT165547 |
| 5 | V9 R5 | *Flavobacterium* | MT165551 |
| 5 | V9 R6 | *Achromobacter* | MT165553 |
| 5 | V9 R7 | *Flavobacterium* | MT165552 |
| 5 | V9 R8 | *Pseudomonas* | MT165528 |
| 5 | V9 R9 | *Stenotrophomonas* | MT165529 |
| 5 | V9 R10 | *Pseudomonas* | MT165530 |
| 5 | V9 R12 | *Pseudomonas* | MT165531 |
| 5 | V9 R14 | *Pseudomonas* | MT165532 |
| 5 | V9 R16 | *Pseudomonas* | MT165533 |
| 5 | V9 R17 | *Stenotrophomonas* | MT165534 |
| 5 | V9 R18 | *Ochrobactrum* | MT165557 |
| 5 | V9 R20 | *Achromobacter* | MT165554 |
| 5 | V9 R21 | *Pseudomonas* | MT165535 |
| 5 | V9 R23 | *Pseudomonas* | MT165536 |
| 5 | V9 R24 | *Achromobacter* | MT165555 |
| 5 | H9 R1 | *Planococcus* | MT165558 |
| 5 | H9 R2 | *Pantoea* | MT165559 |
| 5 | H9 R10 | *Chryseobacterium* | MT165561 |
| 5 | H9 R12 | *Bacillus* | MT165548 |
| 5 | H9 R13 | *Chryseobacterium* | MT165562 |
| 5 | H9 R15 | *Pantoea* | MT165560 |
| 5 | H9 R16 | *Bacillus* | MT165549 |
| 5 | H9 R17 | *Chryseobacterium* | MT165563 |
| 5 | H9 R22 | *Rhizobium* | MT165564 |
| 5 | H9 R24 | *Pseudomonas* | MT165537 |
| 5 | V10 R5 | *Pseudomonas* | MT165538 |
| 5 | V10 R7 | *Paenibacillus* | MT165565 |
| 5 | V10 R9 | *Pseudomonas* | MT165539 |
| 5 | V10 R10 | *Pseudomonas* | MT165540 |
| 5 | V10 R11 | *Paenibacillus* | MT165566 |
| 5 | V10 R12 | *Stenotrophomonas* | MT165570 |
| 5 | V10 R13 | *Paenibacillus* | MT165569 |
| 5 | V10 R14 | *Paenibacillus* | MT165567 |
| 5 | V10 R15 | *Stenotrophomonas* | MT165571 |
| 5 | V10 R17 | *Paenibacillus* | MT165568 |
| 5 | V10 R18 | *Agrobacterium* | MT165578 |
| 5 | V10 R19 | *Bacillus* | MT165550 |
| 5 | V10 R22 | *Pseudomonas* | MT165541 |
| 5 | V10 R23 | *Pseudomonas* | MT165542 |
| 5 | V10 R24 | *Pseudomonas* | MT165543 |
| 5 | H10 R1 | *Stenotrophomonas* | MT165572 |
| 5 | H10 R2 | *Pseudomonas* | MT165544 |
| 5 | H10 R3 | *Pseudomonas* | MT165545 |
| 5 | H10 R7 | *Stenotrophomonas* | MT165573 |
| 5 | H10 R8 | *Pseudomonas* | MT165546 |
| 5 | H10 R10 | *Stenotrophomonas* | MT165574 |
| 5 | H10 R13 | *Achromobacter* | MT165556 |
| 5 | H10 R15 | *Stenotrophomonas* | MT165575 |
| 5 | H10 R21 | *Stenotrophomonas* | MT165576 |
| 5 | H10 R24 | *Stenotrophomonas* | MT165577 |
